# Glucocorticoid receptor regulates protein chaperone, circadian clock and affective disorder genes in the zebrafish brain

**DOI:** 10.1242/dmm.050141

**Published:** 2023-10-02

**Authors:** Helen Eachus, Lara Oberski, Jack Paveley, Irina Bacila, John-Paul Ashton, Umberto Esposito, Fayaz Seifuddin, Mehdi Pirooznia, Eran Elhaik, Marysia Placzek, Nils P. Krone, Vincent T. Cunliffe

**Affiliations:** ^1^ School of Biosciences, University of Sheffield, Firth Court, Western Bank, Sheffield S10 2TN, UK; ^2^ Department of Oncology and Metabolism, Medical School, University of Sheffield, Beech Hill Road, Sheffield S10 2RX, UK; ^3^ Bioinformatics and Computational Biology, National Heart, Lung and Blood Institute, National Institutes of Health, Building 12, 12 South Drive, Bethesda, MD 20892, USA; ^4^ Department of Medicine III, University Hospital Carl Gustav Carus, Technische Universität Dresden, 01307 Dresden, Germany

**Keywords:** DNA methylation, Glucocorticoid, Nervous system, Transcriptome

## Abstract

Glucocorticoid resistance is commonly observed in depression, and has been linked to reduced expression and/or function of the glucocorticoid receptor (NR3C1 in human, hereafter referred to as GR). Previous studies have shown that GR-mutant zebrafish exhibit behavioural abnormalities that are indicative of an affective disorder, suggesting that GR plays a role in brain function. We compared the brain methylomes and brain transcriptomes of adult wild-type and GR-mutant zebrafish, and identified 249 differentially methylated regions (DMRs) that are regulated by GR. These include a cluster of CpG sites within the first intron of *fkbp5*, the gene encoding the glucocorticoid-inducible heat shock protein co-chaperone Fkbp5. RNA-sequencing analysis revealed that genes associated with chaperone-mediated protein folding, the regulation of circadian rhythm and the regulation of metabolism are particularly sensitive to loss of GR function. In addition, we identified subsets of genes exhibiting GR-regulated transcription that are known to regulate behaviour, and are linked to unipolar depression and anxiety. Taken together, our results identify key biological processes and novel molecular mechanisms through which the GR is likely to mediate responses to stress in the adult zebrafish brain, and they provide further support for the zebrafish GR mutant as a model for the study of affective disorders.

## Teaser

**Summary:** This study identifies molecular pathways through which the glucocorticoid receptor (GR) mediates stress responses in the zebrafish brain, providing further support for GR-mutant zebrafish as an affective disorder model.

## INTRODUCTION

The dynamic interactions between an organism and its environment can modify phenotypes by modulating gene expression across the life course. The central nervous system (CNS) integrates sensory information about the external environment with information about the internal physiology of the body to maintain homeostasis. Perception or anticipation of stressful stimuli can elicit a wide range of adaptive responses in animals, including heightened cognition, mobilisation of energy stores and the activation of locomotor responses (McEwen, 2012). These changes can mitigate the adverse impacts of an environmental stressor and enable physiological set points to be restored once exposure to a stressful stimulus has been curtailed. The combination of adaptive physiological responses to behavioural stressors that maintain or restore homeostasis is known as allostasis, a process that is coordinated within the mammalian CNS by interactions between the limbic system and the hypothalamic–pituitary–adrenal (HPA) axis (McEwen and Wingfield, 2010). Stress-induced activation of the HPA axis causes the adrenal gland to release glucocorticoid hormones, such as cortisol or corticosterone, into the circulation, which coordinate allostasis throughout the body. In vertebrates, the primary mediator of glucocorticoid signalling in response to stressors is the glucocorticoid receptor (NR3C1 in human, hereafter referred to as GR), a sequence-specific DNA-binding transcription factor, the transport of which from the cytoplasm to the nucleus is regulated by binding its glucocorticoid ligand (Kadmiel and Cidlowski, 2013). Glucocorticoid-bound GR promotes allostasis by regulating a wide array of target genes involved in metabolism and behaviour, which includes repression of genes that function within the hypothalamus and pituitary gland – such as pro-opiomelanocortin (*POMC*) and corticotrophin-releasing hormone (*CRH*) – in order to attenuate HPA axis activity once a stressful stimulus has subsided (de Kloet et al., 2005). This negative feedback ensures that sustained activity of the HPA axis is limited, contingent on sustained exposure to stressor, and terminated rapidly once exposure to stressor ends.

Although effective allostasis enables coping under stress and the development of resilience, under circumstances of chronic stress or trauma, this effectiveness may be weakened, leading to increased risks of behavioural and physiological dysfunction (Pariante and Lightman, 2008; Reul et al., 2015). Altered expression of the GR-encoding gene *NR3C1*, and of GR target genes, has previously been implicated in the pathobiology of affective disorders in human studies and in animal models (Turecki and Meaney, 2016). A prominent theme emerging from this research is that epigenetic changes, such as altered DNA methylation and/or histone modifications in the vicinity of *NR3C1* and GR target genes, and the accompanying transcriptional changes, are linked to chronic persistence of HPA axis activity, blunted stress responses, loss of mental health and more-rapid ageing (Turecki and Meaney, 2016; Zannas and Chrousos, 2017). However, the molecular mechanisms through which the GR regulates transcriptional activity of target genes, and the roles of epigenetic modifications associated with these genes in the developing and adult brain are still not well understood.

Previous studies in zebrafish have demonstrated a requirement for GR function in the regulation of social behaviour, glucocorticoid signalling, and transcription of HPA axis-specific genes during larval and adult stages (Facchinello et al., 2017; Griffiths et al., 2012; Ziv et al., 2013). Roles for zebrafish GR have been identified in a growing range of biological processes (see Dinarello et al., 2020). These processes include response to hypoxia (Marchi et al., 2020), development of muscle, cartilage and bone (Jiang et al., 2020; Palstra et al., 2018), regulation of circadian rhythm (Morbiato et al., 2019; Mosser et al., 2019; Muto et al., 2013), inflammation (Chatzopoulou et al., 2016; Facchinello et al., 2017; Gans et al., 2020), and in growth and metabolism (Faught and Vijayan, 2019a,b, 2020). However, the impacts of GR function on transcriptional and epigenetic mechanisms in the adult zebrafish brain remain unknown. To investigate these processes, we carried out comparative DNA methylome and transcriptome analyses of the brains of adult male wild-type and GR-mutant zebrafish exhibiting robust behavioural abnormalities, including freezing (i.e. when the fish was immobile), altered light–dark preference and altered boldness/exploratory behaviour. Our results identified groups of functionally related GR-regulated genes that are linked to a small number of highly significant Gene Ontology (GO) terms including protein chaperone, circadian clock and affective disorder-related genes. We showed that the first intron of one of these genes, *fkbp5*, contains a cluster of cytosine and guanine dinucleotides separated by a phosphate (CpGs) that overlaps with putative transcription factor binding sites, the methylation of which in the adult zebrafish brain is regulated by GR function. Moreover, we find that the human orthologues of genes for which transcription is sensitive to loss of GR function in the zebrafish brain are significantly associated with unipolar depression and anxiety disorders. Together, our results provide further support for the GR mutant as a model for the study of affective disorders.

## RESULTS

### Loss of GR function leads to altered methylation of genes in the adult zebrafish brain

**Fig. 1.**
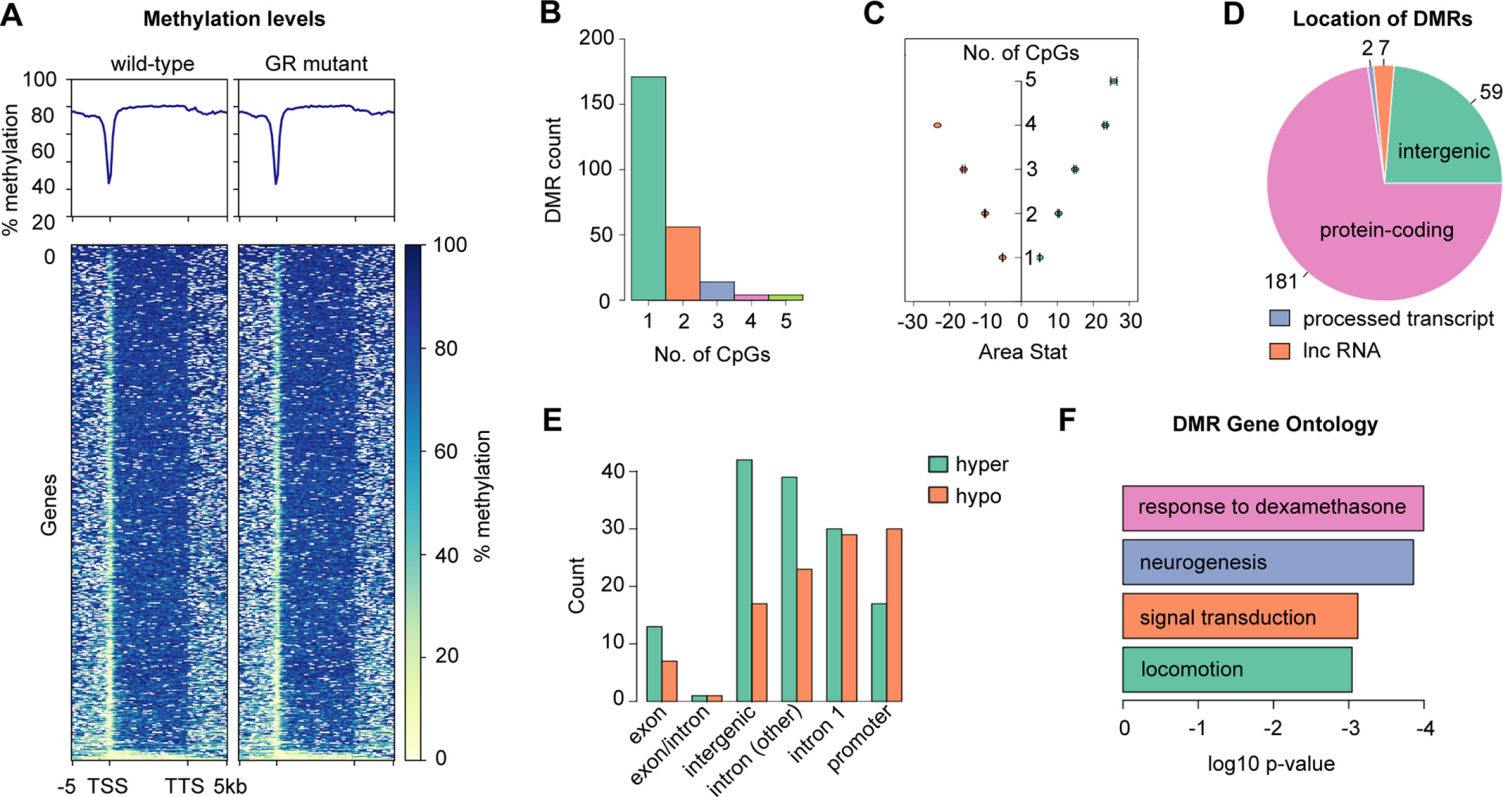
**Whole-genome bisulphite sequencing (WGBS) analysis of wild-type and GR-mutant adult brain samples.** (A) Graphs (top) indicating average DNA methylation (in %) across gene bodies and flanking sequences extending −5 kb upstream of the transcription start site (TSS) and +5 kb downstream of transcription termination site (TTS), in wild-type and GR-mutant adult brain samples analysed by WGBS. Heatmap (bottom) showing the distribution of methylated cytosine and guanine dinucleotides separated by a phosphate (CpGs) within gene bodies and flanking DNA sequences in wild-type and GR-mutant adult brain samples analysed by WGBS, covering the region from −5 kb upstream of the TSS to 5 kb downstream of the TTS, for the 1448 genes for which there was >95% coverage of CpGs within the plotted region. Deeper blue corresponds to a higher level of methylation. In wild-type (*n*=2) and GR*-*mutant (*n*=2) samples, gene bodies are typically highly methylated and promoters exhibit reduced or absent methylation, confirming that the global genomic DNA methylation patterns in the zebrafish brain samples analysed are similar to those previously described. (B) Histogram showing frequency distribution of DMRs identified by WGBS according to the number of CpGs within each DMR. Detailed summaries of all DMRs are provided in Table S1. (C) Plot of the number of CpGs within DMRs against the AreaStat measure of differential methylation (mean±s.e.m.). Negative values indicate DMRs that are hypomethylated and positive values indicate DMRs that are hypermethylated in GR-mutant adult male brain compared to that in wild-type fish. (D) Pie chart indicating the location of DMRs in relation to types of gene. The majority (181 of 249) DMRs are associated with protein-coding genes, some are intergenic, and a small number are associated with long non-coding RNAs (lncRNAs) or processed transcripts. (E) Histogram displaying the number of hypermethylated and hypomethylated DMRs associated with different locations in relation to genes. 47 DMRs are located within a putative gene promoter and 59 are located within the first intron. 22 DMRs are within an exon or a region that potentially pertains to an intron of one gene and an exon of another gene (labelled as exon/intron, whilst 62 DMRs are associated with an intron other than intron 1). (F) The top four slimmed (identified) Gene Ontology (GO) terms associated with biological processes enriched in GR-mutant versus wild-type methylome data. Identification was with the Princeton GO term finder, with a cut-off of *P*<0.05.

Altered patterns of epigenetic modifications, such as DNA methylation, have been implicated as mediators of altered transcriptional responses to behavioural stressors (Gray et al., 2017; Zannas and Chrousos, 2017). To identify DNA methylation changes in the adult zebrafish brain that are caused by mutations in the *nr3c1* gene encoding the GR, we sequenced the whole brain methylomes of two adult wild-type and two adult GR-mutant males aged 13 months. By using the whole-genome bisulfite sequencing (WGBS) approach (von Meyenn et al., 2016), GR-sensitive differentially methylated regions (DMRs) were identified within the zebrafish genome. The HiSeq sequencing run yielded a total of 470,880,704 reads over the four brain samples, of which 95.2% were high-quality reads, and 49.3% of these were mapped to the Zv10 reference genome. Bisulfite conversion to detect non-methylated cytosines was successful for 99.15% of the sequence. The average coverage for all CpGs clusters across the genome was 3.475. The average methylation level across the genome was 84.24% for wild-type and 83.97% for GR-mutant brains. A heatmap of methylation levels across genes exhibiting >95% CpG coverage indicated strong methylation across gene bodies, intermediate methylation of the 5′ and 3′ flanking regions, and reduced or absent methylation at gene promoters (Fig. 1A), confirming that the global genomic DNA methylation patterns in the analysed samples were similar to those described in other vertebrates (Gao et al., 2021; Zhu et al., 2021). By using the bsseq software, we identified 249 loci that exhibit differential methylation in the brain of GR-mutant males compared with that of wild-type males (Table S1). Of the 249 DMRs identified by WGBS analysis, 171 comprised a single CpG site, with an additional 55 DMRs comprising two CpGs, and the remaining 23 DMRs containing three or more clustered CpGs (Fig. 1B). The DMRs identified by WGBS analysis were ranked by using the areaStat parameter, i.e. the sum of the test statistics of all CpG sites within a DMR, such that a larger AreaStat is more likely to indicate a DMR. Indeed, greater AreaStat values were associated with DMRs comprising larger number of CpGs (>3), whereas smaller AreaStat values exhibited a smaller number of CpGs (Fig. 1C). We found that 142 DMRs (57%) exhibited hypermethylation of CpGs within the DMR, and the remaining 107 DMRs (43%) were hypomethylated in the GR-mutant samples compared to wild-type brain samples. Each DMR was annotated with information about their proximity to transcription units (Table S1). Of the 249 DMRs, seven were not close to any gene. Meanwhile, there were five genes (ENSDARG00000093369, *enox2*, *plpp1a*, *slc16a13*, *sil1*), each of which were associated with two separate DMRs, and an additional 16 DMRs were associated with two separate genes. Thus, the 249 DMRs were associated with 253 unique ENSEMBL IDs. Two of the 249 DMRs identified by WGBS were associated with a processed transcript, seven with a long non-coding RNA, 59 were intergenic, and 181 were associated with a protein-coding gene (Fig. 1D). Forty-seven DMRs were located within a putative gene promoter (up to 2 kb upstream of the TSS) and 59 were within the first intron of the corresponding gene (Fig. 1E). Both promoter and first intron sequences are associated with gene regulation. Twenty-two DMRs were located within a DNA sequence that might function as an intron of one gene and an exon of another gene (labelled as exon/intron, Fig. 1E), and 62 DMRs were associated with an intron other than intron 1. Interestingly, when comparing hypomethylated and hypermethylated DMRs, we found a higher number of hypermethylated DMRs in intergenic regions, whereas the number of hypomethylated DMRs was higher in promoter regions (Fig. 1E). GO analysis of the 253 genes associated with the 249 identified DMRs indicated enrichment of genes associated with a response to dexamethasone, neurogenesis, signal transduction or locomotion (Fig. 1F; Table S2).

**Fig. 2.**
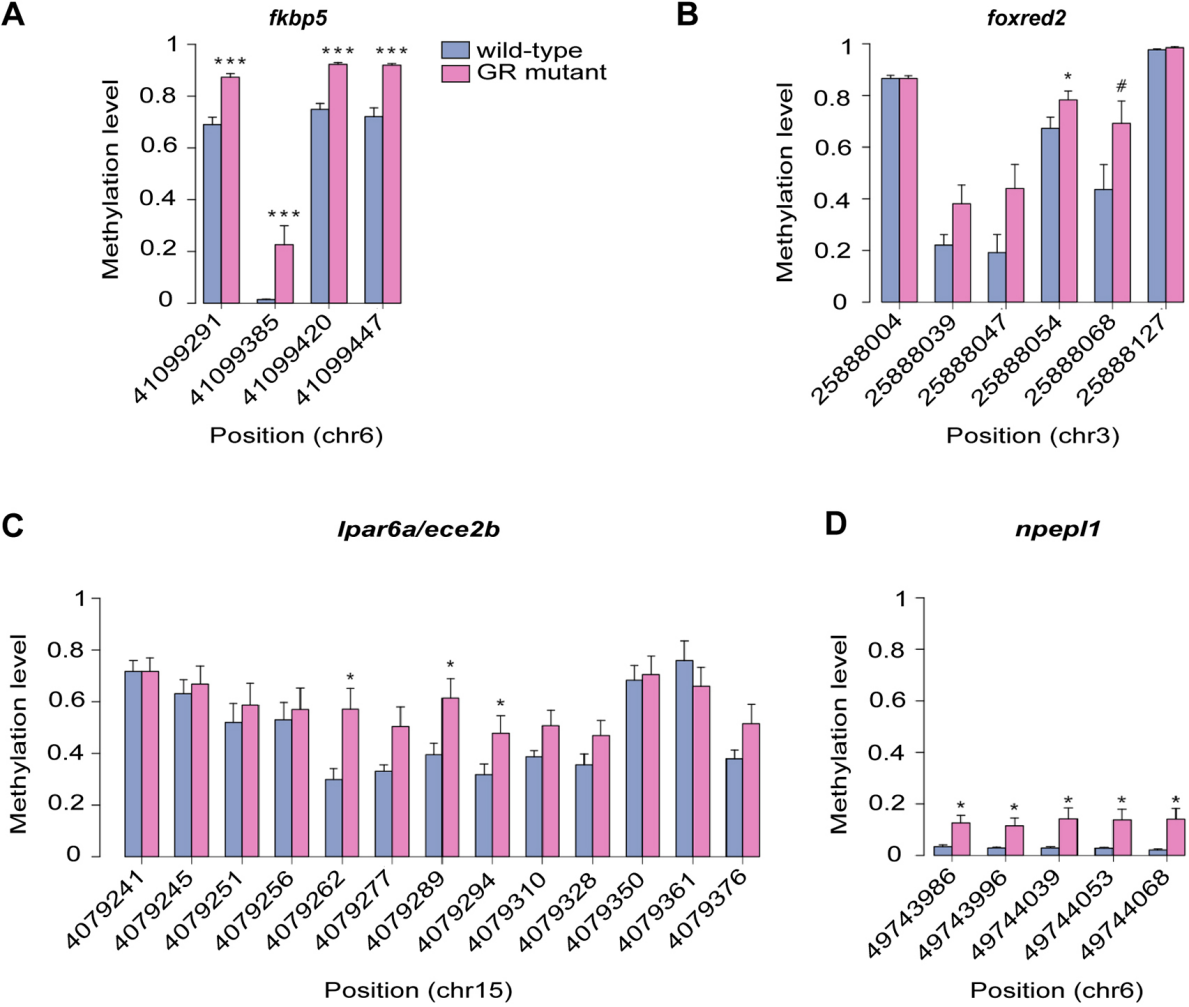
**Targeted analysis of DNA methylation within high-ranking DMRs by using the BisPCR^2^ technique.** (A–D) The percentage of methylation of individual CpGs within a subset of high-ranking DMRs identified in the WGBS analysis was determined using the BisPCR^2^ technique in wild-type (*n*=10) and GR-mutant (*n*=10) adult male zebrafish brains. DMRs associated with the following genes were confirmed by BisPCR^2^: *fkbp5* (A), *foxred2* (B), *lpar6a* (intron 2)*/ece2b* (intron 8) (C), *npepl1* (D); indicated on the *x*-axes is the number of the first nucleotide of each CpG on the respective chromosome of the *Danio rerio* reference genome (GRCz10 release 85). Histograms show mean percentage of methylation±s.e.m. in wild-type and GR-mutant adult brains. On average, a coverage of 4093 reads per amplicon was obtained across all samples subjected to BisPCR^2^ analysis. Significant differences between wild-type and GR-mutant brains regarding the level of CpG methylation were determined using the Mann–Whitney test (^#^*P*<0.06, *P<0.05,***P<0.001).

To confirm that the observed differential methylation of CpGs is mediated by loss of GR function, we selected a subset of 11 high-ranking DMRs within or close to genes – some of which have biologically salient functions (Table S1) – for confirmatory deep sequencing analysis in ten additional wild-type and ten GR-mutant adult male brains, using the BisPCR^2^ technique (Bernstein et al., 2015). Of the 11 DMRs that were subjected to this analysis, four exhibited significant differential methylation in wild-type and GR*-*mutant adult brains (Fig. 2; Fig. S1). The BisPCR^2^ amplicons included additional CpGs within or adjacent to the DMR detected in the original WGBS data set, which provided additional information regarding the size and extent of differential methylation at the DMR that may not have been detected by WGBS due to the limited depth of coverage. One DMR detected by WGBS was located within *fkbp5* – a gene encoding an HSP90-associated protein chaperone known to tether GR in the cytoplasm – and comprised two CpGs within intron 1, which were hypermethylated in the GR-mutant brain. This DMR was confirmed by BisPCR^2^, and two further CpGs were also found to be hypermethylated within the same region (Fig. 2A). WGBS also detected a DMR comprising five CpGs within a cluster of six CpGs upstream of the TSS for *foxred2*, which encodes an FAD-dependent oxidoreductase. Of those five CpGs, two were confirmed to be hypermethylated in the GR-mutant brain by BisPCR^2^ (Fig. 2B). One DMR comprising four CpGs located within intron 2 of *lpar6a* and/or intron 8 of *ece2b* was found by WGBS to be hypermethylated in the GR-mutant brain (Table S1), *lpar6a* encoding a lysophosphatidic acid receptor and *ece2b* an endothelin-converting enzyme. Of those four CpGs, only one was confirmed to be significant in the BisPCR^2^ analysis (4079262), but two additional adjacent CpGs were also found to be significantly hypermethylated in GR-mutant brain by BisPCR^2^ (Fig. 2C). Finally, a DMR comprising three CpGs was detected by WGBS in intron 1 of *npepl1*, a gene encoding an aminopeptidase-like protein (Table S1). These CpGs were confirmed by BisPCR^2^ to be hypermethylated in GR-mutant brains (49744039, 49744053, 49744068; Fig. 2D); two adjacent CpGs (49743986, 49743996) were also found to be hypermethylated (Fig. 2D). Using this technique, the remaining seven DMRs that had been subjected to BisPCR^2^ analysis were not confirmed to be differentially methylated in GR-mutant brains (Fig. S1).

### GR regulates the expression of genes involved in chaperone-mediated protein folding, regulation of circadian rhythm and regulation of metabolism in the zebrafish adult brain

**Fig. 3.**
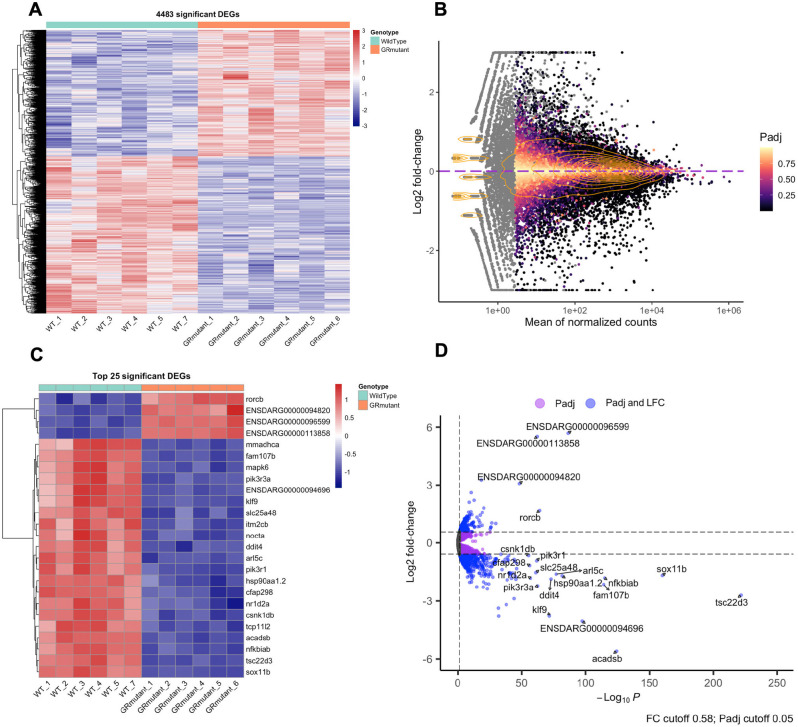
**Differential gene expression in wild-type and GR-mutant brain transcriptomes of adult zebrafish.** (A) Heatmap with hierarchical clustering of genes (rows) showing relative expression of all significant differentially expressed genes (DEGs) (*P*<0.05) in individual wild-type (*n*=6, cyan) and individual GR*-*mutant (*n*=6, orange) brains. Red indicates upregulation, blue indicates downregulation. (B) MA plot with overlaid density contour lines showing individual mean normalised counts for each transcript across all samples plotted against log2 fold-change (LFC) in average counts for each transcript between wild-type and GR-mutant adult brain samples. As the average mean of normalised counts increases, the requirements of a gene to be differentially expressed are more likely to be met. Grey points correspond to genes that were independently filtered from the analysis (high dispersion, low mean of normalised counts) and lack an adjusted *P*-value (Padj). The *P*adj threshold for statistically significant differential expression was set at *P*<0.05 and genes exhibiting significant differential expression are indicated by coloured points. A total of 4483 genes exhibit significant differential expression. Of those, 2001 genes are upregulated and 2482 are downregulated in GR-mutant brains compared to those of wild-type fish. (C) Heatmap with hierarchical clustering of genes (rows) showing relative expression of the top 25 most significant DEGs in individual wild-type (cyan) and GR*-*mutant (orange) brains. Of these genes, four are upregulated and 21 are downregulated in GR*-*mutant brains. (D) Volcano plot showing DEGs in transcriptomes of wild-type and GR*-*mutant adult brain. The statistically significant −log10 *P*adj values are plotted on the *x*-axis against LFC in counts for each transcript (magnitude of differential expression) on the *y*-axis. Dashed lines correspond to *P*adj and LFC cut-off values set at *P*<0.05 and 0.58, respectively. Pink dots indicate DEGs (*P*adj<0.05); blue dots indicate DEGs (*P*adj<0.05) above or below the set LFC threshold of +0.58 or −0.58, respectively. Genes with the 20 smallest *P*adj values, i.e. those with a statistically most-significant differential expression, are named.

We next sought to determine whether changes to the transcriptome could be detected in adult GR-mutant brains. To do this, we performed bulk RNA-sequencing (RNA-Seq) of six adult wild-type and six adult GR-mutant male brains, and sequenced at an average depth of ∼20-30 million reads per sample using a paired-end read strategy. Principal component analysis (PCA) of RNA-Seq data were then combined with hierarchical clustering analysis to investigate transcriptomic similarities between samples. Plotting the first two principal component (PC1 and PC2) values of the RNA-Seq data from the six replicates of wild-type and homozygous GR-mutant adult male brain samples showed that the GR-mutant and the wild-type samples clustered separately (Fig. S2A). This was confirmed by a cluster dendrogram and a hierarchical clustering heat map of RNA-Seq data (Fig. S2B,C). The wild-type and GR*-*mutant brain RNA-Seq library sizes were broadly similar (Fig. S2D), enabling a differential expression analysis of adult wild-type and GR-mutant brain RNA samples by using an adjusted false discovery rate (FDR) of *P*<0.05 to identify genes exhibiting differential expression in the brain samples of the two different genotypes. We identified 4483 differentially expressed genes (DEGs), with expression of 2001 genes increased and that of 2482 decreased in GR-mutant brain samples compared to those of wild-type (Table S3). Hierarchical clustering of genes revealed genotype segregation (Fig. 3A), and an MA plot illustrates the wide variation in the relationship between log2 of fold-changes in expression and overall expression level for all genes (Fig. 3B). Fig. 3C indicates the 25 genes with the most significant differential expression in GR-mutant brain samples compared to wild-type brains. Four of these genes exhibit increased transcription in GR-mutant brain samples, whereas 21 of these genes are downregulated in the GR mutant. A volcano plot of the differential expression data (Fig. 3D) shows log2 fold-changes in transcript abundance against −log10 adjusted *P*-value. Overall, the number of downregulated genes is higher, and they exhibit a higher degree of statistical significance and larger fold-changes than the upregulated genes. The 20 genes with most significant changes in expression (i.e. the smallest adjusted *P*-value; Fig. 3D) include those that encode the transcription factors *tsc22d3*, *sox11b*, *nfkbiab, klf9*, *rorcb* and *nr1d2a*, and the protein chaperone *hsp90aa1.2*, as well as components of intracellular signalling cascades – such as *arl5c*, *pik3r3a, pik3r1*, *csnk1db* – and interactors with components of the cytoskeleton or mitochondria – such as *cfap298*, *ddit4*, *fam107b*, *slc25a48* and *acadsb.*

To identify genes that exhibit both differential methylation and differential expression, we compared the statistically significant genes identified in the methylome and transcriptome data sets. We found that 57 of the 253 DMR gene IDs (22.5%) are included in the transcriptome data set (Fig. S3). We performed a permutation test, using the regioneR package in R, which indicated that there is a significant enrichment in the DMR list for DEGs (Z=2.468, *P*=0.014, *n*=1000 permutations). Of the DMRs we validated in using BisPCR^2^ analysis, we found that *fkbp5* and *foxred2* were both associated with a hypermethylated region, and significantly downregulated in GR-mutant brains (Table S3). *lpar6a* was found to be associated with a hypermethylated region and its transcription was significantly upregulated in GR*-*mutant brains (Table S3). *npepl1* and *ece2b* were associated with a hypermethylated region but neither gene was differentially expressed in GR-mutant brains.

**Fig. 4.**
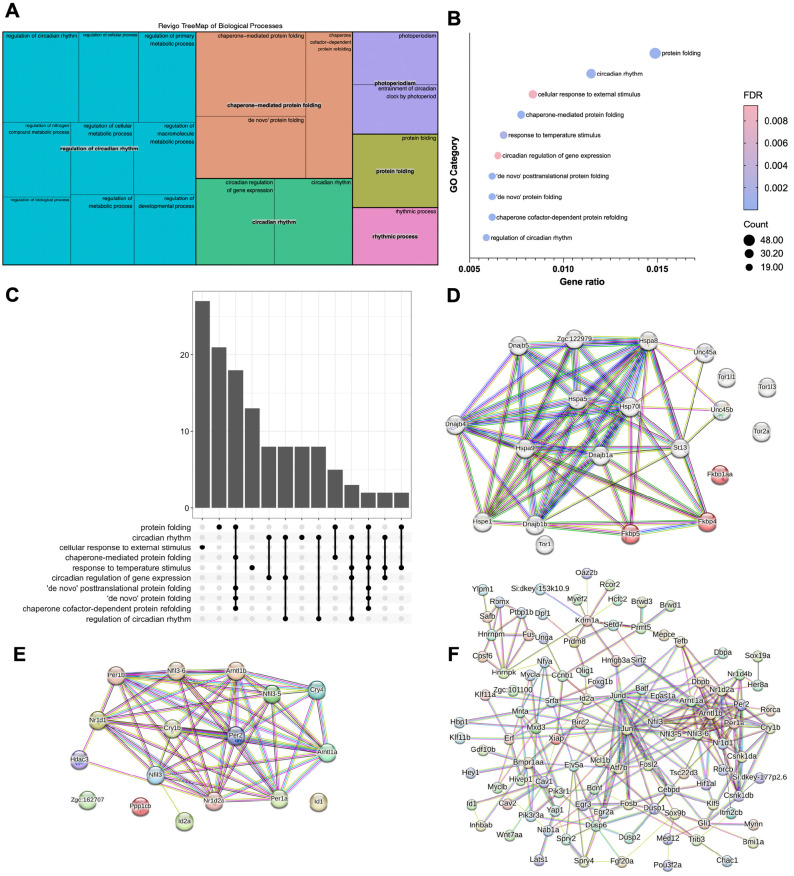
**Gene Ontology and protein–protein interaction network analysis of genes exhibiting differential expression in wild-type and GR-mutant brains.** (A) REVIGO TreeMap produced with a REVIGO-generated R script by using the Biological process terms identified with the Gene Ontology (GO) enrichment analysis and visualisation (GOrilla) tool (threshold *P*-value set to <10^−5^), which are linked to transcripts exhibiting differential expression in wild-type and GR-mutant adult brain transcriptomes (*n*=6 fish, for each genotype). (B) Dot Plot using the enrichGO function within the clusterProfiler R package to identify Biological process GO terms linked to transcripts exhibiting differential expression in wild-type and GR-mutant adult brain transcriptomes (q-value cut-off was 0.05). The circle diameter indicates the number of genes linked to a specific GO term (count number). The gene ratio is the percentage of the total number of differentially expressed genes linked to a specific GO term. (C) UpSet Plot using the enrichGO function within the clusterProfiler R package to identify intersections between Biological process GO term-linked gene sets that exhibit differential expression in wild-type and GR-mutant adult brain transcriptomes. Indicated on the *y*-axis are the number of genes found in the GO categories. (D–F) STRING protein–protein interaction networks of proteins encoded by GR-regulated genes under the GO terms ‘chaperone-mediated protein folding’ (D), ‘circadian rhythm’ (E) or ‘regulation of primary metabolic process’ (F), respectively indicating interactions between many protein chaperones, circadian clock transcription factors or circadian clock transcription factors and immediate-early transcription factors. Individual proteins are nodes, edges are links between proteins signifying the various interaction data that support the interactions, all colouring is according to evidence type (see von Mering et al., 2007) and STRING website for colour legend.

To gain insight into the biological function of GR in regulating the composition of the adult brain transcriptome, GO analysis was conducted to identify the most significant Biological process GO terms linked to DEGs. Setting a stringent statistical significance threshold of *P*<10^−5^, we found a total of 19 Biological process GO terms strongly associated with GR-regulated genes. Of those, the most significant were related to aspects of protein folding, regulation of circadian rhythms and regulation of metabolism (Table S4). The relative significance of these Biological process GO terms was visualised in a REVIGO TreeMap (Fig. 4A) (Supek et al., 2011), and in clusterProfiler (Fig. 4B) using the enrichGO function for over-representation analysis (Wu et al., 2021). In addition, an UpSet plot illustrates the overlap in gene sets that perform similar biological functions between GO categories (Fig. 4C). Of the Biological process GO terms identified by this analysis, the most significant term is ‘chaperone-mediated protein folding’ with 20 DEGs; the second-most significant is ‘circadian regulation of gene expression’ with 14 DEGs (Table S4). Other GO terms closely related to ‘chaperone-mediated protein folding’ with *P*<10^−5^ are ‘*de novo* protein folding’, ‘chaperone co-factor-dependent protein refolding’, ‘*de novo* post-translational protein folding’, and ‘protein folding’. GO terms closely related to ‘circadian regulation of gene expression’ with *P*<10^−5^ are ‘circadian rhythm’, ‘regulation of circadian rhythm’, ‘rhythmic process’, ‘photoperiodism’ and ‘entrainment of circadian clock by photoperiod’. We also identified a third group of five GO terms with *P*<10^−5^, which is related to metabolic regulation, i.e. ‘regulation of primary metabolic process’, ‘regulation of nitrogen compound metabolic process’, ‘regulation of cellular metabolic process’, ‘regulation of macromolecule metabolic process’ and ‘regulation of metabolic process’ (Table S4). The remaining three GO terms with *P*<10^−5^ are ‘regulation of cellular process’, ‘regulation of biological process’ and ‘regulation of developmental process’.

When we investigated the GO category Molecular function, setting a stringent threshold of statistical significance at *P*<10^−5^, GR-regulated genes with a significant adjusted *P*-value were associated with only four GO terms (Table S5): ‘heat shock protein binding’, ‘protein serine/threonine kinase activity’, ‘transcription regulator activity’ and ‘unfolded protein binding’. Of those, heat shock protein binding was the most significant. Taken together, these results indicate that, in the adult brain, the most significant function of GR is to regulate the expression of genes that (1) encode components of ‘chaperone-mediated protein folding’ mechanisms, and (2) are involved in the regulation of circadian rhythms and metabolic processes, such as specific protein kinases and transcription factors. The significance of these interactions was further investigated using STRING to analyse functional protein–protein interactions between members of the GO terms ‘chaperone-mediated protein folding’, ‘circadian rhythm’ and ‘regulation of primary metabolic processes’ (Fig. 4D–F). Plot analysis of proteins associated with chaperone-mediated protein-folding DEGs from our data suggests that most of these proteins are part of a network involving robust interactions between members (Fig. 4D). This is indicated by the strength of the lines between the individual protein nodes, and also apparent for proteins associated with transcription regulatory components of the circadian clock (Fig. 4E), downstream of GR function. Although the number of proteins within the GO term ‘regulation of primary metabolic processes’ was much higher than those in GO terms ‘chaperone-mediated protein folding’ and ‘circadian rhythm’, only a subset of these protein components exhibited particularly robust network interactions (Fig. 4F). The latter were mostly transcription factors, such as Fosb, Jun and Jund, as well as components of the circadian clock (see above and Fig. 4F).

### Altered expression of behaviour-associated genes in the GR-mutant brain

**Fig. 5.**
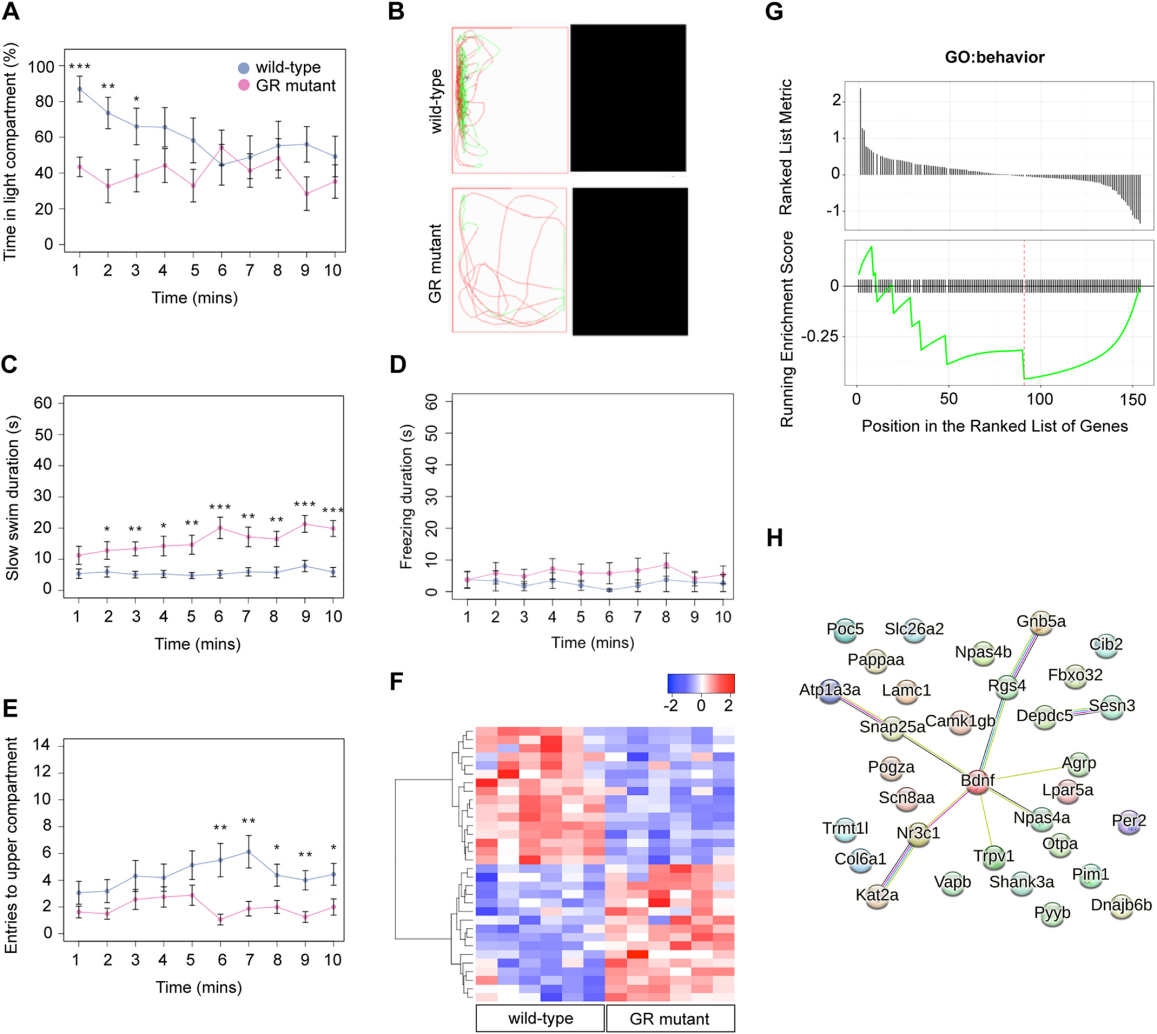
**The glucocorticoid receptor regulates behaviour and expression of behaviour-associated genes in the adult zebrafish.** (A,B) Behavioural analysis of adult fish in the scototaxis test. Plotted in A is the time fish spent in the light part of the tank. Wild-type adults display a significant initial preference for the light part of the tank, whereas GR mutants show no preference for either the light or the dark areas [repeated measures ANOVA, main effect of genotype: *F*=4.8, degrees of freedom (d.f.)=1, 15 (*P*=0.044); main effect of time: *F*=5.53, d.f.=1, 15 (*P*=0.033); genotype: time interaction: *F*=4.60, d.f.=1, 15 (*P*=0.048)]. *n*=8 wild-type fish, *n*=9 GR-mutant fish, one experiment). Asterisks indicate statistical differences in Tukey post-hoc analysis. *P<0.05, **P<0.01, ***P<0.001. (B) Representative traces of wild-type and GR-mutant fish swimming during the first minute of the scototaxis test. Black rectangular areas (right) indicate the dark part of the tank where swim trajectory could not be traced; white rectangular areas (left) indicate the light part of the tank, with fish trajectories indicated in red (fast swimming >9cm/s) and green (medium swimming 2–9 cm/s). (C–E) Behavioural analysis of adult fish during the novel tank diving test. Plotted is the duration of slow swimming (<2 cm/s) (C), and the duration of freezing (D) and exploratory behaviour (E) of adult wild-type (blue) and GR-mutant (red) fish after diving into a novel tank (*n*=16 fish per group, across two independent experiments). (C) Adult GR*-*mutant fish swam slower in the novel tank than wild-type adults [ANOVA with repeated measures: significant effect of genotype: *F*=17.4, d.f.=1, 30 (*P*=0.0002); main effect of time: *F*=25.3, d.f.=1, 286 (*P*≤0.0001); significant genotype: time interaction: *F*=14.3, d.f.=1, 286 (*P*=0.0002)]. *P<0.05, **P<0.01, ***P<0.001. (D) Compared with wild-type fish, GR-mutant fish show no differences in the duration of freezing behaviour in the novel tank diving test [ANOVA with repeated measures: main effect of genotype: *F*=1.087, d.f.=1, 30 (*P*=0.305); main effect of time: *F*=0.05, d.f.=1, 286 (*P*=0.83); genotype: time interaction: *F*=0.35, d.f.=1, 286 (*P*=0.56)]. (E) Adult GR-mutant fish were less exploratory than wild-type adults in the novel tank, exhibiting fewer entries to the upper compartment of the novel tank than wild-type fish [ANOVA with repeated measures: main effect of genotype: *F*=7.38, d.f.=1, 30 (*P*=0.011); main effect of time: *F*=1.9, d.f.=1, 286 (*P*=0.169); genotype: time interaction: *F*=4.84, d.f.=1, 286 (*P*=0.0286)]. Asterisks indicate significant difference in Tukey post-hoc analysis. *P<0.05, **P<0.01, ***P<0.001. All behaviour plots show mean±s.e.m. (F) Heatmap showing relative expression (normalised counts) of 32 genes associated with GO term ‘behavior’, which are differentially expressed in the wild-type and GR-mutant adult brain transcriptomes. These DEGs included equal numbers of upregulated and downregulated genes. (G) Gene set enrichment analysis (GSEA) plots of all genes associated with the GO term ‘behavior’ detected in the RNA-Seq data set. (Top panel) Distribution of the fold-change through the list of genes. (Bottom panel) Plotted is the running sum (green line) of the enrichment scores, with the red dashed line indicating the maximum enrichment score for the gene set. The row of black vertical lines indicates the positions of individual genes. The enrichment score for GO term ‘behavior’ was −0.458 (*P*=0.035), suggesting significant enrichment of behaviour-associated DEGs within this dataset. (H) STRING protein–protein interaction network of proteins encoded by GR-regulated genes in the GO category Biological process under the term ‘behavior’ indicates roles for Bdnf and for functional interactors, such as Agrp, Npas4a, Trpv1, Snap25a and Rgs4 downstream of GR function. Individual proteins are shown as nodes, edges are links between proteins signifying the various interaction data that support the interactions, all colouring is according to evidence type (see von Mering et al., 2007) and STRING website for colour legend.

Previous studies have demonstrated that adult GR-mutant fish exhibit a range of behavioural abnormalities consistent with an affective disorder (Ziv et al., 2013). Accordingly, blood cortisol concentrations are considerably elevated in both larvae and adults (Ziv et al., 2013; Griffiths et al., 2012). We confirmed that, compared with their wild-type siblings, GR-mutant adult fish swam slower in the open field, exhibited reduced thigmotaxis (i.e. swimming close to the walls of their holding tank), displayed increased freezing behaviours (i.e. when the fish was immobile) and had high levels of whole-body cortisol (Fig. S4A–D). We further observed that mutant fish exhibited reduced dark aversion in a scototaxis test (Fig. 5A,B). The novel tank diving test can be used to measure novelty-induced anxiety-associated behaviours, such as erratic movements and reduced exploratory swimming. In this test, GR-mutants still swam slower than wild types, which may indicate a lack of fast erratic movements. However, although there was no difference in the duration of freezing behaviour in this test (Fig. 5C,D), GR mutants also made fewer entries to the upper area of the tank in the novel tank diving test (Fig. 5E), which may be indicative of reduced boldness or reduced exploratory behaviour. Taken together, our confirmatory and novel findings of behavioural abnormalities GR mutants indicate that in the adult brain, GR function is required to regulate behaviour.

We next sought to test whether our transcriptomic data include genes that are known to regulate behaviour, and identified 32 significant DEGs associated with the GO term ‘behavior’ (Fig. 5F). We also performed gene set enrichment analysis (GSEA) (Subramanian et al., 2005) of the 117 genes associated with the GO term ‘behavior’ detected in our transcriptomic dataset and observed a significant enrichment of behaviour-associated DEGs (Fig. 5G). A STRING analysis to identify functional protein–protein interactions between GR-regulated proteins under ‘behavior’ in the GO category Biological process revealed relatively few interactions (Fig. 5H). However, Bdnf was implicated in multiple functional interactions with Agrp, Npas4a, Trpv1, Snap25a and Rgs4 proteins, suggestive of an important role for Bdnf signalling downstream of GR function to regulate behaviour.

**Fig. 6.**
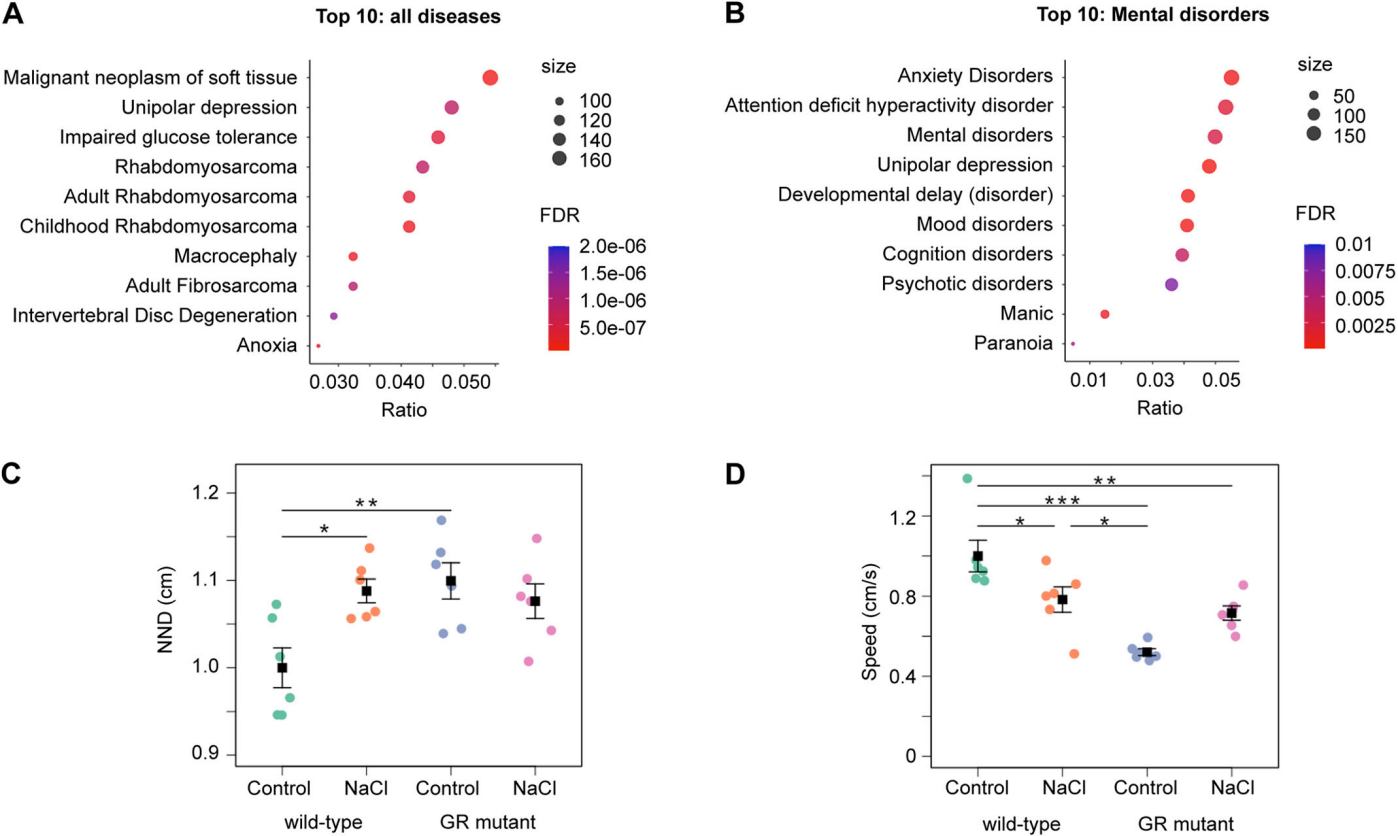
**The glucocorticoid receptor regulates depression-associated genes in adult zebrafish brain and stress-responsive locomotor behaviour in zebrafish larvae.** (A) Enrichment analysis to identify diseases associated with human orthologues of GR-regulated genes by using the disgenet2r package to search the DisGeNET database. Human orthologues of zebrafish genes exhibiting significant differential expression in wild-type and GR*-*mutant adult brain samples were analysed using all data available in this database. A total of 678 diseases, symptoms and syndromes are significantly associated with orthologues of GR-regulated zebrafish genes; an adjusted false discovery rate (FDR) cut-off of *P*<0.05 was used (Table S6). The top ten diseases were selected based on FDR and then ranked according to the gene ratio. Unipolar depression ranked second after malignant neoplasm of soft tissue. (B) The top ten diseases within the Mental Disorders category of the DisGeNET database were identified using the disgenet2r package, selected based on FDR and then ranked according to the gene ratio. Anxiety disorders ranked first and unipolar depression fourth. (C,D) Group swimming analysis of zebrafish larvae at 5 days post fertilization under baseline and stressed conditions for nearest neighbour distance (NND) and swim speed. Panel C shows that the NND of wild-type groups increases under stress, whereas mutant groups have a higher NND than wild-type groups under baseline conditions and their NND is not affected by stress [two-way ANOVA followed by Tukey post-hoc test; genotype: treatment interaction, *F*=8.09, d.f.=1, 20 (*P*=0.01)]. Panel D shows that the swimming speed of wild-type groups is reduced under saline stress (NaCl), whereas GR-mutant groups swim slower than wild-type fish under baseline conditions and their swim speed is not affected by NaCl-induced stress [two-way ANOVA followed by Tukey post-hoc test; genotype: treatment interaction, *F*=14.41; d.f.=1, 20 (*P*=0.001)]. Wild-type and GR-mutant groups (both *n*=12) across two independent experiments. Plots show the mean±s.e.m. *P<0.05, **P<0.01, ***P<0.001.

It has previously been noted that the behavioural abnormalities of the zebrafish GR mutant, which have been shown to be ameliorated by treatment with the antidepressant fluoxetine, are reminiscent of an affective disorder (Ziv et al., 2013). To explore this relationship further, we carried out an enrichment analysis using the full list of 4483 genes exhibiting differential expression in wild-type and GR-mutant adult brain samples for the presence of orthologues of human disease genes, using the disgenet2r package to search the DisGeNET database. A total of 678 diseases, symptoms or syndromes were associated with these DEGs (adjusted FDR, *P*<0.05; Table S6). Ranking by gene ratio indicated that unipolar depression comes second after malignant neoplasm of soft tissue in the list of diseases most-significantly associated with genes exhibiting differential expression in wild-type and GR-mutant adult brain samples (Fig. 6A). Moreover, when disgenet2r enrichment analysis was specifically focused on the mental disorders category (Mental Disorders, Disease Class F03), 26 disorders were significantly associated with the DEGs (adjusted FDR, *P*<0.05; Table S7). Anxiety disorders ranked first and unipolar depression ranked fourth in this analysis (Fig. 6B).

One of the more consistent physiological phenotypes associated with affective disorders is hyperactivity of the HPA axis (Pariante and Lightman, 2008). Indeed, we observed hypercortisolaemia in GR-mutant larvae and adults (Fig. S4D,E), suggesting that dysregulated HPA axis activity, which could alter behavioural stress responses, develops early during embryonic or larval stages in the GR mutant. To explore this possibility, we analysed group swimming behaviour of wild-type and GR-mutant larvae under unstressed and saline-stressed conditions (Fig. 6C,D). When wild-type larvae were exposed to acute saline stress, we found an increase in their nearest neighbour distance (NND) and a decrease in their swim speed. By contrast, unstressed GR-mutant larvae exhibited an NND that was no different to that of saline-stressed wild-type larvae and did not change after saline stress (Fig. 6C). Moreover, compared with saline-stressed wild-type larvae, unstressed GR-mutant larvae exhibited a reduced swim speed that remained low after saline stress (Fig. 6D). Thus, unstressed GR-mutant larvae exhibit constitutive behavioural abnormalities that are similar to the changes induced in wild-type larvae after exposure to an acute stressor.

### The co-chaperone gene *fkbp5* exhibits differential methylation and differential expression in gr^s357^ mutant zebrafish brains

**Fig. 7.**
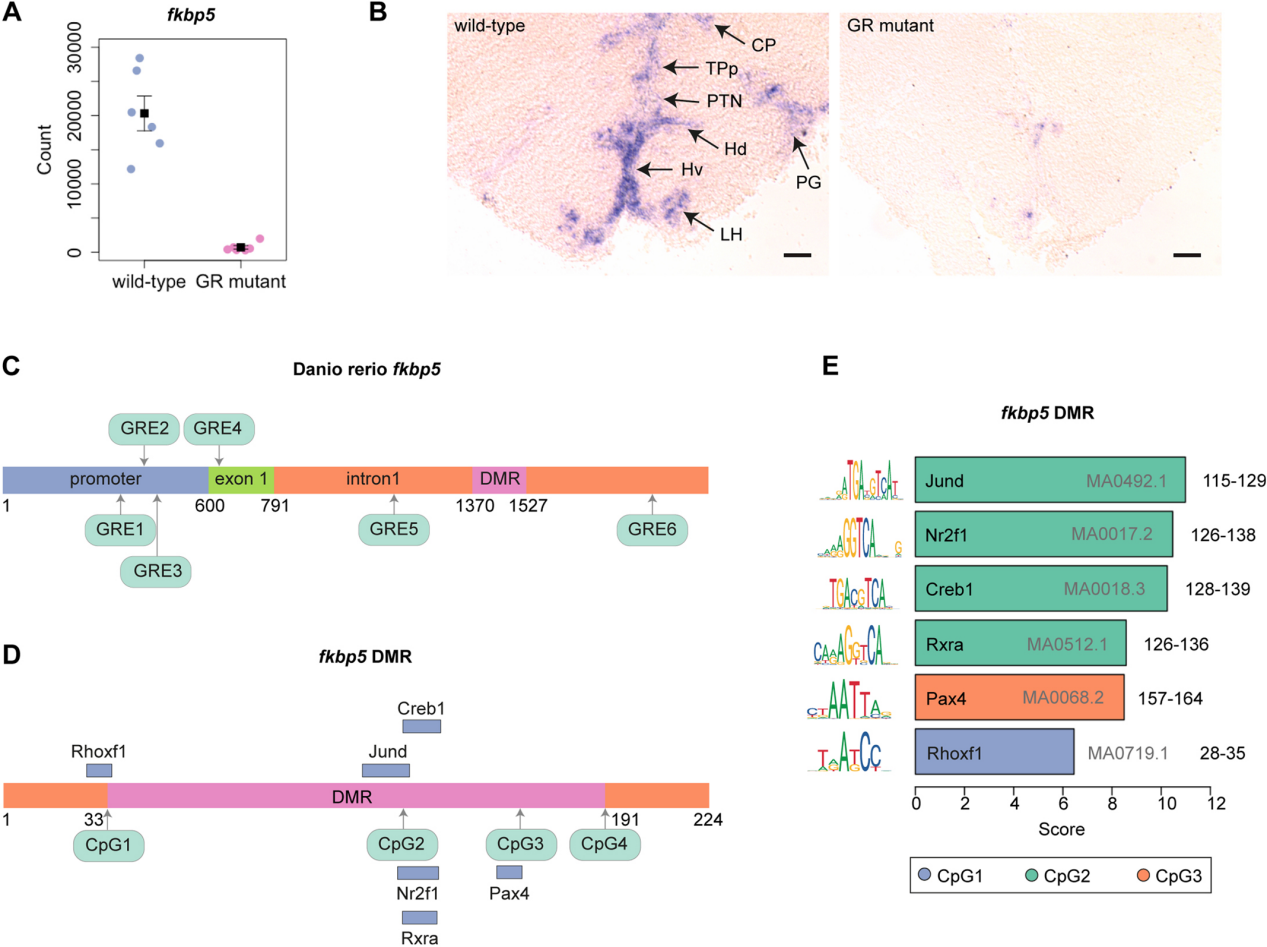
***fkbp5* is differentially expressed and differentially methylated in wild-type and GR-mutant zebrafish brains.** (A) GR function is required for *fkbp5* expression in the adult brain. Normalised transcript-count data for *fkbp5* obtained from RNA-Seq analysis of wild-type and GR-mutant fish (both *n*=6) adult brains (*P*=3.45×10^−25^). (B) GR function is required for *fkbp5* expression in the adult brain. *In situ* hybridisation analysis of *fkbp5* transcripts in transverse sections of adult zebrafish brain, cross section 149 (Wulliman et al., 1996) from wild-type (left panel) and GR-mutant (right panel) adult males, at a rostro-caudal position through the thalamus (CP, central posterior thalamic nucleus), the posterior tuberculum (TPp, periventricular nucleus of posterior tuberculum; PTN, posterior tuberal nucleus; PG, preglomerular nucleus) and the hypothalamus (Hd and Hv, dorsal and ventral zone of periventricular hypothalamus, respectively; LH, lateral hypothalamic nucleus). Results show *fkbp5* expression in wild-type brain that is almost completely extinguished in the GR-mutant brain. Wild-type and GR-mutant (both *n*=6) fish, across three independent experiments. Scale bars: 100 μm. (C) Schematic of *fkbp5*, showing promoter region, exon 1 and intron1. Six distinct GR-binding sites (GREs) within the promoter region (GRE1, GRE2, GRE3), exon 1 (GRE4) and intron 1 (GRE5, GRE6) are shown in green*.* (D) Schematic of the differentially methylated region (DMR; pink) within *fkbp5*, surrounded either side by two 33-bp-long flanking sequences (orange). Four cytosine and guanine dinucleotides separated by a phosphate, i.e. CpG1, CpG2, CpG3, CpG4, are shown in green (see Fig. 2A for chromosome 6 nucleotide coordinates). Binding sites for transcription factors Rhoxf1, Jund, Nr2f1, Rxra, Creb1 and Pax4 overlap with CpGs, and are shown as blue bars. (E) Bar graph of data obtained by using the JASPAR database, indicating that CpG1 of the *fkbp5* DMR is located within the binding motif for transcription factor RHOXF1, CpG2 is located within the binding motifs for transcription factors Creb1, Jund, Nr2f1 and Rxra, and CpG3 is located within the binding motif for transcription factor Pax4. Location of CpG4 is not located within a known transcription factor-binding motif. JASPAR matrix IDs are shown in grey within or to the right of each bar. DNA coordinates (nucleotide numbers) for each CpG within the *fkbp5* intron 1 as depicted in panel D, are shown in black. The consensus sequence for the DNA-binding motif is shown to the left of each bar.

Of the four GR-regulated DMRs confirmed by our BisPCR^2^ analysis, three are linked to genes exhibiting differential expression in wild-type and GR*-*mutant adult brain samples, i.e. *fkbp5* (DEG ranking: 87/4483, adjusted *P*-value=8.96×10^−23^), *foxred2* (DEG ranking: 840/4483, adjusted *P*-value=1.88×10^−5^) and *lpar6a* (DEG ranking: 1114/4483, adjusted *P*-value=1.30×10^−4^). Given its relatively high DEG ranking, regulation of *fkbp5* was investigated further. The robust hypermethylation of the *fkbp5* DMR in GR-mutant adult brain samples was accompanied by a substantial reduction in *fkbp5* transcript abundance, as confirmed by the plotting of read count data extracted from the RNA-Seq analysis (Fig. 7A). *In situ* hybridisation on wild-type and GR-mutant brain transverse sections by using an *fkbp5* RNA probe revealed that a specific pattern of *fkbp5* expression was extinguished in the thalamus, posterior tuberculum and hypothalamus of the GR-mutant brain (Fig. 7B).

Sequence analysis of the *fkbp5* promoter revealed the presence of three glucocorticoid response elements (GREs) within a 200 bp region located 100-300 bp upstream the transcription start site, one GRE within exon 1 and a further two GREs in intron 1 (Fig. 7C). Sequence analysis of the *fkbp5* intron 1 DMR indicates close similarities of the sequences spanning three of the four differentially methylated CpGs within this DMR (Fig. 7D) to binding sites for transcription factors Jund, Nr2f1, Creb1, Rxra, Pax4 and Rhoxf1 (Fig. 7E). Thus, it is possible that activated GR bound to one or more of the GREs in the *fkbp5* promoter regulates recruitment of these transcription factors to the *fkbp5* intron 1 DMR and/or demethylation of the CpGs within their putative binding sites.

## DISCUSSION

In this study, we combined genome-wide methylome and RNA-Seq analyses of brains from adult wild-type and GR-mutant zebrafish to identify genomic targets of the GR transcription factor. Our results indicate that, in response to stress-induced glucocorticoid signalling, primary functions of GR in the brain are to boost expression of an extensive network of genes linked to the GO term ‘chaperone-mediated protein folding’ and regulate the expression of components of the circadian oscillator network. GR mutants also display robust behavioural abnormalities in a variety of tests, and they exhibit differential expression of behaviour-associated genes. Integrative analysis of our results, using the DisGeNET platform to identify human gene–disease associations, further revealed that human orthologues of those zebrafish genes whose transcription is sensitive to loss of GR function are significantly associated with unipolar depression and anxiety disorders. Together, our results identify novel molecular mechanisms through which GR is likely to mediate responses to stress in the adult zebrafish brain and provide support for the GR mutant as a model of affective disorders.

### GR-mediated DNA methylation in the adult zebrafish brain

Our methylome analyses of the zebrafish genome, comparing wild-type and GR-mutant fish, has identified CpGs for which methylation is regulated by GR function in the adult zebrafish brain. The DMRs identified by WGBS are linked to genes encoding components of the response to dexamethasone, implying that some of them are linked to known GR-regulated genes, as well as an association with genes associated with neurogenesis. In rodents, neurogenesis has been linked to regulation of behaviour, including anxiety-associated behaviours (Aimone et al., 2014), suggesting that altered regulation of neurogenesis due to loss of GR function contributes to the affective-like behaviours also observed in this mutant.

The majority of identified DMRs were associated with protein-coding genes, where they are mostly located within a putative promoter region or first intron in which *cis*-regulatory elements are frequently located. Many DMRs are located in intragenic regions, where methylation has also been shown to modulate gene expression (Keller et al., 2016). Our BisPCR^2^ experiments confirmed that four of the 11 DMRs identified by WGBS exhibited robust DNA methylation differences in whole-brain samples of wild-type and GR-mutant fish. Thus, of the DMRs identified by WGBS, only a third were validated through BisPCR^2^. The unvalidated DMRs might represent regions that exhibit a stochastic variation in methylation or sequence reads derived from rare cell types within the brain. To explore these possibilities further, future work could include a deeper WGBS analysis of more adult brain samples, and identification of more GR-regulated DMRs linked to DEGs. Overall, we found significantly fewer DMRs than the number of differentially expressed transcripts identified in the RNA-Seq analysis. This is likely to be due to the small WGBS sample size (*n*=2) per genotype, the relatively low sequencing depth, as well as the tissue complexity and cellular heterogeneity of the adult brain tissue. We found an overlap of 22.5% between the identified GR-regulated DMRs and DEGs. There is an inverse relationship between promoter and/or intron 1 methylation and gene transcription (Anastasiadi et al., 2018). Accordingly, hypermethylation of DMRs for *fkbp5* and *foxred2* is consistent with the significantly downregulated gene expression we observed for these genes in GR mutants. However, methylation also occurs widely in other parts of the genome and has been associated with other functions, such as regulating alternative promoter sites (Maunakea et al., 2010). Accordingly, differential methylation associated with DMRs at non-promoter sites may be associated with functions, such as promoter selection, that could have relatively small effects on transcript abundance.

### GR-mediated regulation of chaperone-mediated protein folding gene expression

Bioinformatic analysis of the RNA-Seq data revealed a small number of Biological process GO terms that, with high statistical significance, were linked to GR function. Of these, ‘chaperone-mediated protein folding’ was the most significant. Consistent with these findings, the most significant GO term in the Molecular function category was ‘heat shock protein binding’. Genes linked to this term included members of the Hsp70 family (Finka et al., 2015), and the Hsp40 family of protein chaperones that stimulate the protein-folding ATPase activity of Hsp70 proteins (Balchin et al., 2020; Kampinga and Craig, 2010). Other genes linked to these GO terms include *st13*, encoding an HSP70-interacting protein that promotes the function of ligand-activated GR (Nelson et al., 2004), and *hspe1*, encoding an Hsp10-like heptameric ring chaperonin that promotes ATP-dependent protein folding in the mitochondrial matrix (Hartman et al., 1992). Tor1, Tor1l1, Tor2a and Tor1l3 are transmembrane-spanning ER and nuclear-envelope-localised AAA+ ATPases, i.e. ATPases associated with a diverse cellular activities, with roles in maintaining integrity and function of the nuclear pore complex and nuclear membrane (Laudermilch and Schlieker, 2016). Unc45a and Unc45b are protein chaperones that form oligomers binding to Hsp90 and Hsp70, and assist in the re-folding of myosin proteins after stress-induced denaturation (Hellerschmied and Clausen, 2014). Fkbp1aa, Fkbp4 and Fkbp5 are members of the peptidyl-prolyl cis-trans isomerase family of co-chaperones, with dual roles in Hsp90-mediated protein folding and protein peptidyl-prolyl isomerisation (Rein, 2020). Hsp90 is a well-described cytoplasmic tether protein for GR that renders the nuclear import of GR cortisol dependent (Grad and Picard, 2007). Furthermore, *fkbp4* and *fkbp5* are glucocorticoid-regulated genes; their protein products are also involved in regulating glucocorticoid-dependent import of GR into the nucleus (Zannas et al., 2016). Taking all these observations into consideration, our results indicate that one primary function of GR in the brain is to systematically boost expression of an extensive network of genes linked to the GO term ‘chaperone-mediated protein folding’ in response to stress-induced glucocorticoid signalling, which could then facilitate behavioural and physiological allostasis. Endocrine responses to environmental stressors are important and well-documented mediators of adaptive phenotypic plasticity (Dennis et al., 2014; Lema, 2020; Sommer, 2020). Moreover, heat shock proteins have been implicated to enable phenotypic plasticity through their ability to refold and remodel protein structure (Zabinsky et al., 2019). It is, therefore, possible that the transcriptomic response to glucocorticoid signalling in the brain includes many genes that encode protein chaperones in order to underpin the neural and behavioural flexibility required for adaptive responses to stressors.

### GR-mediated regulation of genes involved in circadian rhythms

Our GO analysis shows that ‘circadian regulation of gene expression’, ‘circadian rhythm’ and ‘regulation of circadian rhythm’ were among the top four most-significant Biological process GO terms associated with GR function in the zebrafish brain. The circadian clock is a molecular oscillator network generating the rhythms of endocrine and metabolic activity that is needed for modulating physiology and behaviour across the day (Cox and Takahashi, 2019). Studies in mammals and zebrafish have demonstrated that substantial, highly conserved and regulatory cross-talk occurs between the GR and circadian transcription factors (Dickmeis et al., 2007, 2013; Froland Steindal and Whitmore, 2019; Jaikumar et al., 2020; Weger et al., 2016). In mammals, the core loop of the circadian oscillator network is made up of interactions between heterodimers of the CLOCK and BMAL1 transcription factors bound to E-boxes within *Per* and *Cry* target genes (Cox and Takahashi, 2019). PER and CRY proteins attenuate transcription of CLOCK/BMAL1 target genes, such as *Nr1d1* and *Nr1d2* (Cox and Takahashi, 2019), which encode transcription factors that regulate expression of Nfil3 genes (Zhao et al., 2021). The results of our transcriptomic analysis indicate that a major function of GR in the zebrafish brain is to regulate the expression of many components of the circadian oscillator network and its primary target genes, including *arnt1a, arnt1b, cry1b, cry4, cry5, nr1d1, nr1d2a, per1a, per1b, per2, nfil3-5* and *nfil3-6*. However, whether all these genes are directly regulated by GR binding to GREs within their regulatory elements remains to be elucidated. A recent analysis of gene expression of Clock genes in GR-mutant zebrafish larvae did not find any difference in expression of Clock genes including *per1*, *per2*, *clocka* and *nr1d2a* (Jaikumar et al., 2020)*,* all of which were differentially expressed in our analysis. This discrepancy is likely to be due to the difference in tissue and developmental stage, since Jaikumar and colleagues used whole larval samples rather than adult brain. However, in a different GR-mutant line (*gr^ia30/ia30^*) differential expression and differences in the phases of expression peaks of Clock genes were observed both in samples of whole larvae and various tissues of adult GR mutants (Morbiato et al., 2019). The study by Morbiato et al. highlights the tissue specificity of Clock gene expression, but, since this study did not analyse adult brain, it is difficult to make comparisons with our data. Nevertheless, our findings are in broad agreement with their conclusion that a major function of GR is to modulate expression of genes involved in circadian rhythms.

### GR-mediated regulation of genes involved in metabolic processes

A third group of five significant GO terms describes regulation of metabolic pathways. These are ‘regulation of primary metabolic process’, ‘regulation of nitrogen compound metabolic process’, ‘regulation of cellular metabolic process’, ‘regulation of macromolecule metabolic process’ and ‘regulation of metabolic process’. All five of these GO terms are linked to the same eight genes within the top 30 DEGs ranked according to adjusted *P*-values: *tsc22d3*, *sox11b*, *klf9*, *rorcb*, *nr1d2a*, *csnk1db*, *itm2cb* and *foxg1b*. Previous studies of *tsc22d3* and *klf9* or their orthologues in other vertebrates have demonstrated important roles for these transcription factors downstream of GR function (Bonett et al., 2009; Cui et al., 2019; Gans et al., 2020, 2021; Lebow et al., 2019). Intriguingly, mammalian homologues of *csnk1db*, i.e. *Csnk1d*, *Csnk1e* and *Csnk2*, are known to phosphorylate PER1 and thus influence circadian rhythm (Blau, 2008; Lowrey et al., 2000; Xu et al., 2005) and, indeed, both *csnk1db* and *csnk1da* are GR-regulated genes also annotated with the GO term ‘regulation of circadian rhythm’ (Table S4). Similarly, the circadian transcription factor *nr1d2a* is associated with multiple circadian rhythm and regulation of metabolism GO terms (Table S4). Additionally, STRING protein–protein interaction analysis for the GR-regulated proteins in the Regulation of primary metabolic process GO category identified many circadian clock components as robust interactors within this category. Thus, our results imply that the circadian oscillator network is an important glucocorticoid-responsive regulator of metabolic pathways in the adult zebrafish brain, the activity of which is boosted during daylight hours as part of the allostatic response to endocrine stress.

### GR-mediated regulation of genes associated with behaviour and affective disorders

Our behavioural studies of adult zebrafish in the open field confirmed other published results stating that GR mutants exhibit increased freezing behaviour, slow swimming and reduced thigmotaxis, all of which may represent biomarkers of an affective disorder (Ziv et al., 2013). Increased freezing behaviour has been related to an anxiety- or depressive-like behaviour, and reduced thigmotaxis potentially reflects a reduction in motivated exploratory behaviour (Ziv et al., 2013). However, the interpretation of a link between reduced thigmotaxis and anxiety has been contested (Rajput et al., 2022). It has also been suggested that increased time spent around the perimeter of the tank is indicative of anxiety (Kalueff et al., 2013), since this behaviour can be reduced by exposure to anxiolytic drugs (Schnörr et al., 2012). Furthermore, some studies have found that thigmotaxic behaviour is increased in stressed zebrafish (Borba et al., 2023), whilst others have found no effect of stress on this behaviour (Golla et al., 2020).

Nevertheless, the additional behavioural tests we performed further support the suggestion that the altered behaviours of GR-mutant adults are symptomatic of an affective disorder (Ziv et al., 2013). The lack of light preference exhibited by GR mutants in the light–dark preference test, compared to the light preference exhibited by wild-type fish, is reminiscent of other reports of wild-type fish exhibiting a light preference that is absent in animals exposed to stress (Chakravarty et al., 2013; Champagne et al., 2010). Moreover, light avoidance has been proposed to be an anxiety-like behaviour of adult zebrafish (Maximino et al., 2011, 2010) since it is ameliorated by treatment with anxiolytic drugs (Fontana et al., 2022; Maximino et al., 2011). In the novel tank diving test, GR*-*mutant fish swam slower and made fewer entries to the upper compartment of the tank than wild-type fish. This behaviour has also been considered indicative of anxiety since it is reduced by prior stress exposure and increased by administration of anxiolytics (Cachat et al., 2010; Fontana et al., 2022; Golla et al., 2020). As documented previously by Griffiths et al. (2012), we found that GR*-*mutant larvae swim slower than wild-type larvae when observed in a group context. Moreover, group cohesion – as measured by their NND – was much lower in GR*-*mutant compared with wild-type larvae, potentially indicating an impairment in social behaviour. Interestingly, we found that the reduced group cohesion and swim speed of GR*-*mutant larvae were similar to the values exhibited by wild-type fish after exposure to saline stress but did not decrease further upon exposure to saline stress. These observations are consistent with published findings that GR*-*mutant fish cannot modulate levels of cortisol and HPI axis-associated genes following stress exposure (Faught and Vijayan, 2019a; Ziv et al., 2013), and that GR-mutant fish do not habituate to a novel tank after repeated exposure to it (Ziv et al., 2013).

We identified 32 GR-regulated genes associated with the GO term ‘behavior’. These genes include *otpa,* known to play a role in social behaviour (Wircer et al., 2017), as well as *agrp* (Shainer et al., 2019) and *bdnf* (Blanco et al., 2020), which are thought to play roles in feeding behaviour. Since *bdnf* has previously been implicated in regulation of zebrafish circadian rhythmicity (D'Agostino et al., 2022; Lucon-Xiccato et al., 2022), it will be of interest to determine whether its role in this process lies downstream of GR function.

Interrogating the DisGeNET database of disease-associated genes (Piñero et al., 2020) with the list of 4483 GR-regulated genes indicated that genes for which transcription is sensitive to loss of GR function in the brain are significantly associated with both unipolar depression and anxiety disorders. In humans, loss of GR function causes Chrousos syndrome (Nicolaides and Charmandari, 2015), in which HPA axis function is dysregulated, leading to hypercortisolaemia, similar to what we observed in GR-mutant fish. Hypercortisolaemia is one of the most-consistent hormonal characteristics of affective disorders, (Holsboer, 2000; Pariante and Lightman, 2008) and increased methylation of *NR3C1* is accompanied by reduced *NR3C1* transcription in some trauma-related psychiatric illnesses (McGowan et al., 2009; Turecki and Meaney, 2016). Thus, loss of *NR3C1* function and/or expression may increase risk of these psychiatric disorders, and the zebrafish GR mutant may be a useful model in which to elucidate aspects of their pathophysiology.

Epigenetic changes at the human *FKBP5* locus have previously been implicated in PTSD and depression resulting from childhood trauma (Klengel et al., 2013). Exposure to childhood trauma is linked to decreased methylation of CpG dinucleotides within intron 2 of *FKBP5*, increased *FKBP5* transcription and suppression of GR function (Klengel et al., 2013). Our study revealed that the zebrafish *fkbp5* locus exhibited changes in both DNA methylation and gene transcription in the GR-mutant adult brain. We observed a robust hypermethylation of four CpGs within the *fkbp5* intron 1 DMR of GR-mutant brain samples, accompanied by a dramatic reduction in *fkbp5* transcription. The least-methylated CpG within this DMR, CpG2, lies within binding sites for Creb1, Jund, Nr2f1 and Rxra, whilst CpG1 lies within a binding site for Rhoxf1, and CpG3 lies within a binding site for Pax4 (Fig. 7). The almost complete lack of methylation of CpG2 in wild-type adult zebrafish brain suggests that, *in vivo*, it can be bound by one or more of the Creb1, Jund, Nr2f1 and Rxra transcription factors, which could prevent its methylation. Interestingly, *creb1b* was 0.7-fold downregulated, *jund* was 0.6-fold downregulated and *nr2f1a* was 1.25-fold upregulated in the GR mutant compared to wild-type brain samples (Table S4). Thus, the reduced levels of Creb1b and Jund proteins and/or the increased level of Nr2f1a, caused by loss of GR function, could facilitate increased DNA methyltransferase (DNMT) action at CpG2, leading to its hypermethylation in GR-mutant brain samples. In our transcriptomic analysis of the GR-mutant brain, we detected significant upregulation of *dnmt3aa* and *dnmt3bb.3*, zebrafish orthologues of the mammalian ‘*de novo*’ methyltransferases DNMT3A and DNMT3B, respectively (Okano et al., 1999). Upregulation of these DNMTs could facilitate hypermethylation of loci, such as the *fkbp5* DMR in the GR-mutant brain.

As a co-chaperone for Hsp90 and an inhibitor of GR nuclear translocation, Fkbp5 may be an important functional bridge between Hsp90 and GR, regulating a chaperone-based phenotypic plasticity response to endocrine signalling. Accordingly, FKBP5-mediated attenuation of GR function might curtail the psychological and behavioural flexibility that is compromised in people with depression and anxiety (Kashdan and Rottenberg, 2010). Our observation that, unlike wild-type larvae, GR*-*mutant larvae did not modulate their behaviour in response to acute osmotic shock, indicates that GR-mutant zebrafish are unable to switch between behavioural states in response to stress. This phenotype could, at least in part, reflect their hypercortisolaemic state, and might be akin to the loss of psychological and behavioural flexibility in people with affective disorders.

Another important regulator of the stress response is the mineralocorticoid receptor (NR3C2 in human, hereafter referred to as MR), encoded by *nr3c2*, the close relative of *nr3c1*. Indeed, work in zebrafish larvae indicates that both GR and MR are involved in stress-regulation in zebrafish larvae (Faught and Vijayan, 2018). Moreover, MR is required for both initiation and termination of the endocrine stress response, and for the modulation of behaviours, such as thigmotaxis in MR-mutant larvae (Faught and Vijayan, 2018). Interestingly, we observed upregulation of *nr3c2* transcripts in the GR-mutant adult brain (Table S3). Altered expression and/or function of MR might contribute to the phenotypes we observed in the GR mutant, hypercortisolaemia of which could cause MR activation, with impacts on behaviour. Thus, future research, comparing the phenotypes of GR, MR and GR–MR double-mutant zebrafish will deepen our understanding of how transcription factors GR and MR contribute to stress regulation in the brain.

The GR-regulated genes and biological processes identified in this study represent promising subjects for further investigation of how loss of GR function dysregulates gene expression in the brain and, thereby, could contribute to behavioural dysfunction. Furthermore, given the utility of the zebrafish for high-throughput small-molecule screening, our results identify molecular pathways that may include tractable targets for the discovery of new treatments for affective disorders.

## MATERIALS AND METHODS

### Zebrafish husbandry and genotyping of stocks

Adult zebrafish were maintained with a 14 h light/10 h dark cycle at 28°C according to standard protocols and were mated using spawning tanks. The *nr3c1* mutant allele *gr^s357^* (Ziv et al., 2013) was obtained from Herwig Baier (MPI for Neurobiology, Martinsried, Germany). Homozygous mutant and wild-type adult sibling populations were created by in-crossing adult *gr^s357^* heterozygotes, and the resulting larvae were raised to adulthood. Young adults were genotyped by fin-clipping and sequencing of genomic DNA-derived PCR products encompassing the *s357* mutation, to establish groups of homozygous wild-type and homozygous *gr^s357^* mutant fish (which from here onwards will be referred to as GR-mutant fish). Wild-type and GR-mutant fish were then maintained in separate tanks and used for behavioural and molecular analysis. All procedures involving experimental animals were performed under the authority of licences granted by the UK Home Office, in compliance with local and national animal welfare guidelines and in accordance with the UK Animals (Scientific Procedures) Act 1986. Adult zebrafish were analysed aged 11–22 months and age-matched within each experiment. Brains used for WGBS, targeted bisulfite sequencing and RNA-Seq transcriptome analysis as well as adult body samples for steroid extraction were from males, whilst behaviour experiments used both males and females. All tissue sampling was conducted between 10:00 and 12:00 on the day of culling and dissection.

### Behavioural analysis

ZebraLab and Shoaling Software (Viewpoint, France) was used to track the movement of individual zebrafish when isolated and in groups, respectively, to provide quantitative measures of swimming behaviour, as previously described by Eachus et al. (2017). All fish used for behavioural analysis were 11 months of age. The tank used for the open field test and the scototaxis test was 25×15×15 cm and filled to 4.1 l, whereas the trapezoid tank used for the novel tank diving test was 23.5×6.2×13.5 cm and filled maximally, as previously described by Eachus et al. (2017). Fish were transported to the behaviour room on the morning of testing and allowed to acclimatise to the behaviour room for 1 h prior to the experiment to allow any stress-induced (via transportation) cortisol levels to return to baseline. All experiments were carried out in a custom-built soundproof booth with controlled lighting and temperature (28°C).

#### Open field test

Individual fish were acclimatised to the empty tank for 1 h prior to testing, horizontal swimming behaviour was then recorded for 10 min, as previously described by Eachus et al. (2017). Instances of freezing behaviour (i.e. when the fish was immobile) were counted when lasting >1 s. To define the duration of thigmotaxis/perimeter swimming, the tank was divided into an inner and outer compartment, in which the outer compartment consisted of an area around the perimeter of the tank of equal volume to the inner compartment. The open field test utilised five male and four female fish of each genotype.

#### Novel tank diving test

Vertical swimming behaviour of individual fish was recorded and binned at 1-min intervals in a 10-min test, as previously described by Eachus et al. (2017). Slow swimming is defined as any swimming movement of velocity up to 2 cm/s. Fish were acclimatised to the behavioural analysis room for 1 h prior to testing, then placed into the tank used for the novel tank diving whereupon testing began immediately. For the novel tank diving test, nine mutant females and seven mutant males, and eight wild-type females and eight wild-type males were used.

#### Scototaxis test

Horizontal swimming behaviour of individual fish performed in the light half of the tank, was recorded during 10 min and data were binned every 1 min, as previously described by Eachus et al. (2017). Fish were acclimatised to the behavioural analysis room for 1 h prior to testing, then placed into the tank, whereupon testing began immediately. For the scototaxis test, four wild-type male and four wild-type female, and four mutant male and five mutant female fish were used.

#### Group swimming analysis of larvae

Groups of 21 larvae aged 5 days post fertilisation (dpf) were analysed in a standard 90 mm circular Petri dish, filled with 50 ml E3 medium, and horizontal swimming behaviour was then recorded for 10 min, as previously described by Eachus et al. (2017). Groups of larvae were acclimatised to the Petri dishes in the behavioural analysis room for 1 h prior to testing. 12 groups of wild-type and 12 groups of GR-mutant fish were analysed across two independent experiments.

### Extraction and quantification of cortisol

Individual adult zebrafish were culled via a rapid overdose of Tricaine followed by decapitation and the body was snap-frozen for cortisol extraction. Adult fish used for cortisol analysis were aged 20 months. Larval cortisol samples were collected from pools of 21 snap-frozen larvae aged 5 dpf, in an Eppendorf tube. Cortisol was extracted using previously described protocols (Cachat et al., 2010; Yeh et al., 2013). Whole-body cortisol levels were then quantified using the ELISA protocol as previously described (Cachat et al., 2010; Yeh et al., 2013).

### WGBS

Whole brains of wild type and GR mutant (*n*=2 each) were dissected from 13-month-old adult male zebrafish and genomic DNA was extracted using the Qiagen DNAeasy kit. DNA sequencing libraries were prepared and sequenced using 125 bp paired-end reads on an Illumina HiSeq by GATC Biotech. Sequence data quality was inspected using FastQC and reads were trimmed to phred-quality Q15. Reads were aligned to the reference genome (*Danio rerio*, GRCz10 release 85) using Bismark with the default parameters (Krueger and Andrews, 2011). PCR duplicates were removed and methylation calls were made using Bismark v0.15.0. Regions that were differentially methylated in mutants were detected using the R package bsseq (v1.8.2) with default parameters: the data were smoothed, low coverage (<2x coverage in all samples) CpGs were removed, and t-statistics were computed and thresholded at 4.6 to identify differentially methylated CpGs (https://support.bioconductor.org/p/78227/). The WGBS DNA sequence data and associated metadata are available at the NCBI Gene Expression Omnibus (GEO) under accession number GSE120632.

Methylation heatmaps were constructed using DeepTools (v3.1.3). Genes were scaled to 10,000 bp and methylation was averaged in 250 bp bins. Methylation levels were plotted for 1448 genes exhibiting >95% CpG coverage within the interval spanning 5 kb upstream of the transcription start site (TSS), the gene body and 5 kb downstream of the transcription termination site (TTS), for wild-type and GR-mutant samples. GO analysis of 253 ENSEMBL IDs associated with the DMRs was performed using GO Term Finder (Boyle et al., 2004) with a *P*-value cut-off of 0.05. The top ten GO terms were manually identified, i.e. slimmed, according to EBI QuickGO ancestry charts.

### Targeted bisulfite sequencing of amplicons by using the BisPCR^2^ method

Whole brains (from ten wild-type and ten GR-mutant adult male zebrafish aged 15 months) were dissected from terminally anaesthetised adult zebrafish and genomic DNA was extracted using the Qiagen DNAeasy kit. Library preparation was based on previously published protocols (Bernstein et al., 2015). Of each DNA sample, ∼200 ng was bisulfite treated using the Qiagen Epitect kit according to manufacturer's instructions, and quantified using an Agilent Tapestation with High-sensitivity tape.

Bisulfite-converted DNA (100 pg) was amplified for 40 cycles in ‘PCR round 1’ by using KAPA Hifi Uracil+ Readymix with 300 nM primer. The optimum annealing temperature and MgCl_2_ concentration for the primer pairs was determined by analysing PCR products on a 2% agarose gel. The ‘PCR round 1’ primers included a 25-30 bp amplicon-specific sequence, designed using MethPrimer (Bernstein et al., 2015) plus an 18 bp overhang to allow the annealing of ‘PCR round 2’ primers (see Table S8). All ‘PCR round 1’ amplicons were pooled for each individual sample at 10 ng/amplicon based on quantification via the Agilent Tapestation. Excess primers and dNTPs were removed using the Qiagen PCR clean-up kit. Individual samples were then barcoded via a second round of PCR. ‘PCR round 2’ reactions were 20 µl, using the KAPA Hifi Uracil+ mastermix, with 300 nM primer and 1 ng of pooled ‘PCR round 1’ product for each sample. The ‘PCR round 2’ forward primer was identical to the 5′ overhang on the ‘PCR round 1’ amplicons, whilst the reverse primer included a region to anneal to the 3′ overhang on the ‘PCR round 1’ amplicons, plus a 6 bp sample-specific barcode. The final barcoded amplicons ranged from 199-270 bp. Excess primers and dNTPs were removed using the Qiagen PCR clean-up kit. Sample quantity and size were determined using the Agilent Tapestation. Products outside of the expected range were removed using AMPure XP beads. Samples were then pooled in an equimolar manner, and a 500 ng pool was sequenced by GATC Biotech (Ebersberg, Germany). Illumina adapters were ligated by GATC Biotech and the samples were sequenced on the Illumina HiSeq for 150 bp paired-end reads.

Sequence quality was determined using FastQC. Sequence data were demultiplexed using QIIME 1 (Caporaso et al., 2010; Li and Dahiya, 2002) and primers were trimmed using cutadapt software (Martin, 2011). Data were aligned to the reference genome (*Danio rerio*, GRCz10 release 85) and methylation levels were called using BSseeker2 (Guo et al., 2013). DNA sequence data and associated metadata are available at GEO (accession number GSE120632). JASPAR was used to identify transcription factor binding sites within the fkbp5 DMR (Sandelin et al., 2004).

### RNA-Seq transcriptome analysis

Brains were dissected from terminally anaesthetised 22-month-old adult male zebrafish with olfactory bulbs and pituitary glands intact and stored individually at −80°C. Total RNA extraction was performed using the commercial RNAeasy Mini Kit (Qiagen) as per manufacturer's instructions. RNA integrity was evaluated on a 1% TAE agarose gel using the Agilent Bioanalyser (2100, Agilent Technologies, USA). RNA library preparation and sequencing were performed by the Deep Sequencing Facility at Technische Universität Dresden (Dresden, Germany). mRNA isolation was achieved using the NEB Next poly(A) mRNA magnetic isolation module with approximately 300 ng total RNA input. Libraries were produced using the NEBNext Ultra II Directional RNA Library Prep Kit for Illumina (NEB, Ipswich, MA, USA). mRNA libraries were sequenced using a 375 bp paired-end method and performed on an Illumina NovaSeq 6000 system (Illumina, San Diego, CA, USA) to a depth of ∼30 million reads per sample. Raw data sequencing files (Fastq.gz) were produced by Technische Universität Dresden for further analysis. RNA-Seq reads were trimmed using Trimmomatic v0.39 (ILLUMINACLIP:Trimmomatic-0.39/adapters/TruSeq3-PE-2.fa:2:30:10) (Bolger et al., 2014), and rRNA sequence contaminants were removed using SortmeRNA v2.1 (Kopylova et al., 2012). Sequence reads were quality-controlled using FastQC. Individual quality control files were combined with MultiQC (Ewels et al., 2016). Quality-processed sequence reads were aligned to the zebrafish genome (GRCz11) by using STAR software and the number of reads per gene was quantified using its quantMode GeneCounts option (Dobin et al., 2013). These steps were performed on the high-performance computing (HPC) cluster ShARC (University of Sheffield). Bioinformatic analysis of RNA-Seq data was performed using RStudio (R v3.6.1). R package DESeq2 was used for differential expression and statistical analysis (Love et al., 2014). Data were normalised with an in-built function using the median-of-ratios method. Wald test was used for hypothesis testing and Benjamini-Hochberg correction was used to correct for multiple comparisons. The ‘result’ function accepted FDR score input of DESeq2 was 0.05, a more stringent threshold than the default 0.1. The threshold of significance was an adjusted *P*-value <0.05. PCA plots, heatmaps and graphs were created using the ggplot2 package in R.

RNA-Seq data and associated metadata are available at GEO under accession number GSE213505.

Additional threshold-based gene set testing was conducted using clusterProfiler Bioconductor package, Release (3.15) (Wu et al., 2021; Yu et al., 2012). To use its enrichGO function, IDs of significant DEGs, and all genes in the dataset were inputted using org.Dr.eg.db (Genome wide annotation for *Danio rerio*) package to map genes to biological pathways. The q-value cut-off was 0.05. Enrichment analysis of disease-associated genes was conducted using the disgenet2r package (v7.0) and compared to all DisGeNET databases (Piñero et al., 2017, 2015, 2020, 2021). STRING (version 11.5) was used to identify functional protein–protein interaction networks (Snel et al., 2000; Szklarczyk et al., 2021).

### *In situ* hybridisation

Adult brains were dissected from humanely culled fish aged 20 months and fixed in fresh in 4% paraformaldehyde in 0.12 M phosphate buffer (4% PFA PB) overnight at 4°C, and then transferred to 30% sucrose (S0389; Sigma), rocking at 4°C for 1-2 days until they sank. Brains were mounted in OCT (361603E; VWR International) and frozen on dry ice before storage at −80°C. Sequential serial 15 μm-thick coronal sections were cut using a cryostat (Leica Biosystems) and collected directly onto Superfrost Plus slides (Fisher Scientific) and stored at −20°C. Chromogenic *in situ* hybridisation was then performed as previously described (Moore et al., 2022). Colour development was performed for an equal duration for wild-type and GR-mutant brain sections.

### Statistical analysis

Statistical analysis and graphics were created in ‘R’ and GraphPad Prism. Data were tested for equal variance and normality prior to analysis. Statistical significance was tested using *t*-tests or Analysis Of Variance and post-hoc analysis via pairwise comparisons. Data normality was evaluated using multiple normality tests, including Shapiro–Wilk, D'Agostino-Pearson and Kolmogorov–Smirnov. A Tukey multiple comparison test was used post-ANOVA to identify means between which a significant difference was observed. If data did not meet the required assumptions for any statistical test, outliers were identified for exclusion using ROUT method, data transformations or non-parametric tests. Where data under analysis were not normally distributed, significance was determined using the Mann–Whitney test. In all graphs, bars represent the mean±s.e.m. *P<0.05, **P<0.01, ***P<0.001, ****P<0.0001.

## Supplementary Material

10.1242/dmm.050141_sup1 Supplementary information

## First Person

This article has an associated First Person interview with the first author of the paper.
