# Supplementary material for: Glucocorticoid receptor regulates protein chaperone, circadian clock and affective disorder genes in the zebrafish brain
Source: Dis Model Mech. 2023 Oct 2;16(9):dmm050141. doi: 10.1242/dmm.050141 (PMC10565112; doi:10.1242/dmm.050141)
Supplement: Supplementary information [file dmm-16-050141-s1.pdf]

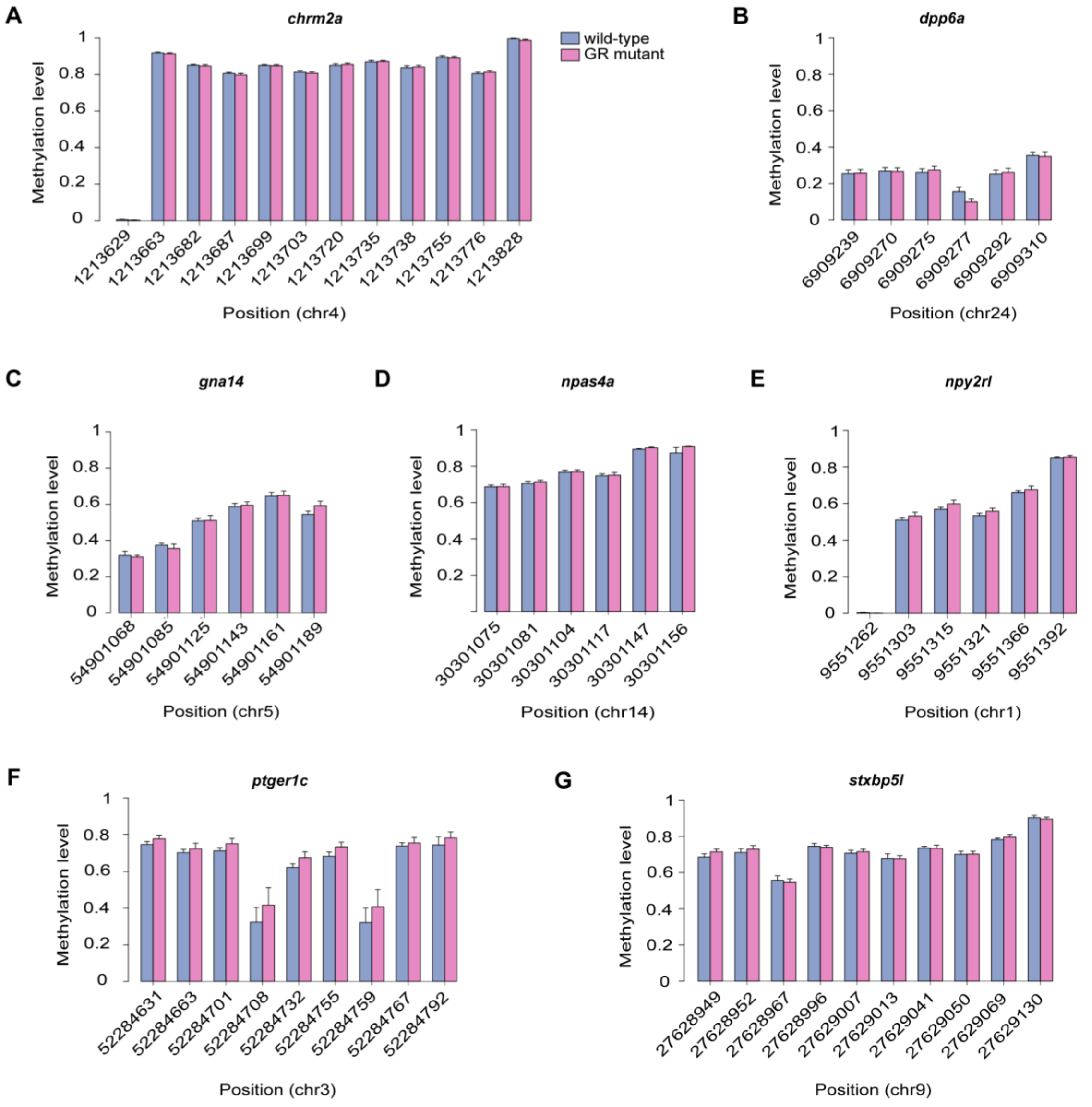

Fig. S1. Additional targeted analysis of DNA methylation, within high-ranking GR-DMRs identified by WGBS, using the BisPCR^2 technique.

(A-G) Percentage methylation of individual CpGs within an additional subset of high-ranking GR-DMRs identified in the WGBS analysis, was determined using the BisPCR^2 technique in 10 wild-type and 10 GR mutant adult male brains: (A) *chrm2a*, (B) *dpp6a*, (C) *gna14,* (D) *npas4a*, (E) *npy2rl*, (F) *ptgerc1,* (G) *stxbp1*. Histograms show mean percentage methylation +/- s.e.m. in wild-type and GR mutant adult brains. On average, a coverage of 4093 reads per amplicon was obtained, averaged across all samples subjected to BisPCR^2 analysis. No significant differentially methylated CpGs were identified in these amplicons using the Mann-Whitney test.

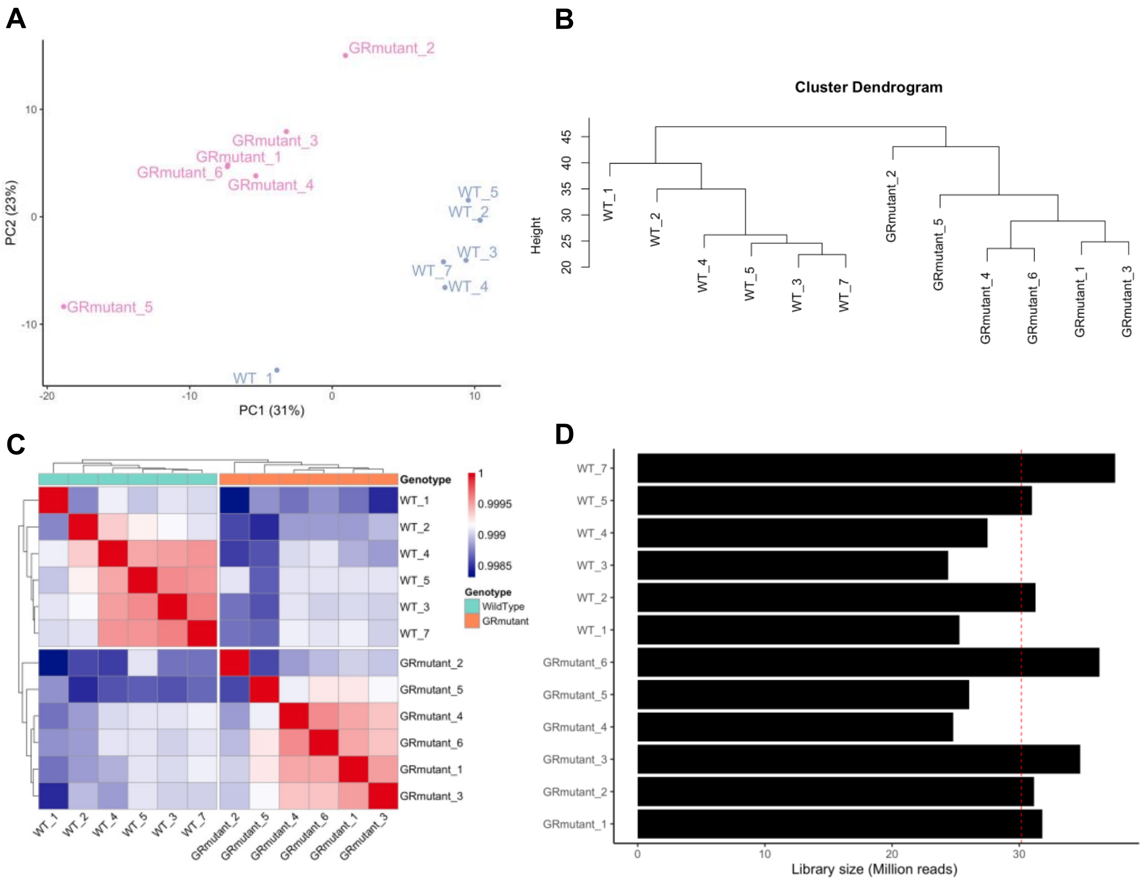

Fig. S2. Transcriptomic analysis of wild-type and GR mutant zebrafish adult brain. Brains were dissected from 22-month old adult males and subjected to RNA-seq analysis using DESeq2 (n=6 fish each). Biological quality control demonstrates that the transcriptomes of wild-type and GR mutant brain samples are significantly different. (A) Principal Component Analysis of RNA-seq data, plotting the first two Principal Components for whole brains from 6 replicates of wild-type (blue) and homozygous GR mutant (pink) adult males demonstrated greater similarity between replicates of the same genotype than between samples of different genotype. (B) Clustering analysis of RNA-seq data confirms that replicate samples of the same genotype exhibited greater similarity to one another than to samples of the other genotype. (C) Hierarchical clustering heat map analysis shows the close degree of similarity between wild-type replicates (n=6) and between GR mutant (n=6) brain

transcriptomes. Overall, the transcriptomes of all wild-type samples clustered together, and WTTL3, WTTL4, WTTL5 and WTTL7 are the most similar wild-type samples. Correspondingly, all GR mutant samples clustered together, and GRMutant1, GRMutant3, GRMutant4 and GRMutant6 are the most similar GR mutant samples. (D) Library sizes (in units of Millions of reads) were as follows: GRMutant1: 31.80427, GRMutant2: 31.17174, GRMutant3: 34.78990, GRMutant4: 24.80914, GRMutant5: 26.05794, GRMutant6: 36.313387, WT1: 25.28883, WT2: 31.28593, WT3: 24.40768, WT4: 27.5044, WT5: 30.99421, WT7: 37.54890.

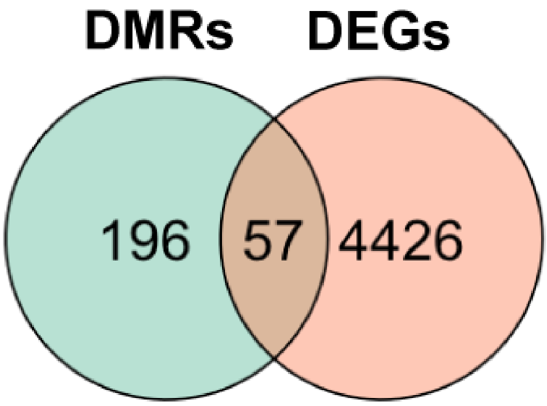

Fig. S3. Overlapping of GR-regulated genes identified in the methylomic and transcriptomic analyses. Venn diagram shows that 57 of 253 (22.5%) DMR gene IDs are included in the transcriptomic data set (DEGs).

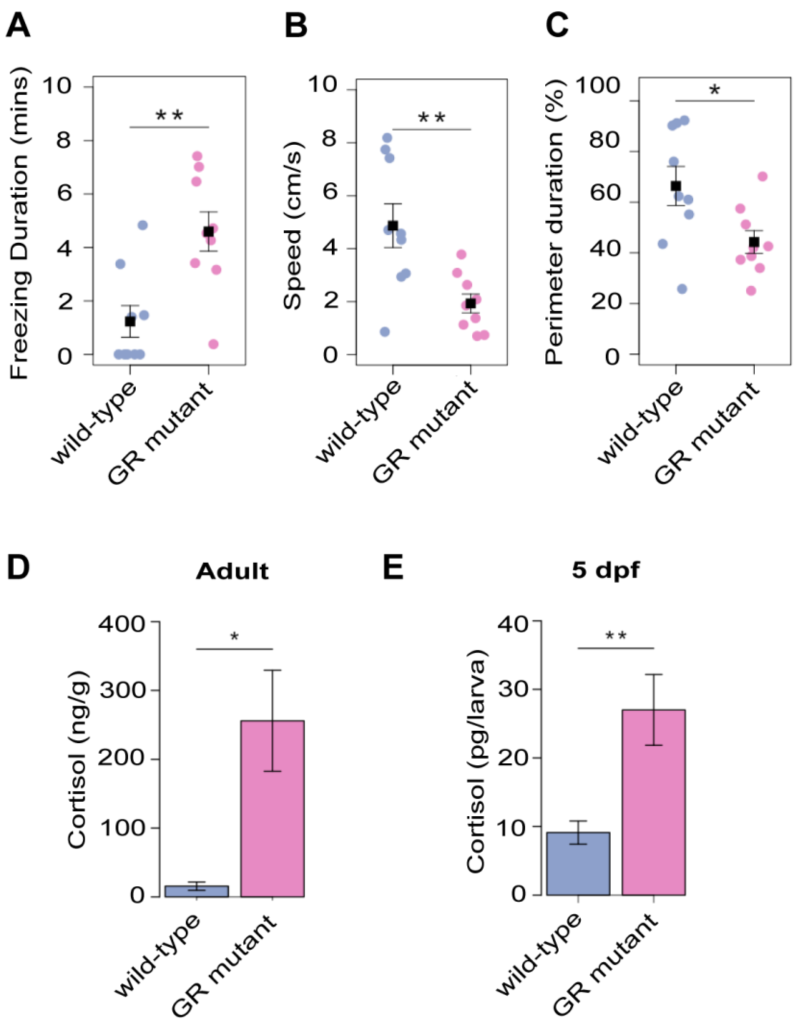

Fig. S4. The Glucocorticoid Receptor (GR) regulates adult zebrafish swimming behaviour in the open field test and whole-body cortisol levels in both larval and adult zebrafish.

(A-C) Behavioural analysis of adult fish in the open field test reveals that GR mutants show (A) increased duration of freezing (two-tailed *t*-test, *t*=-3.57, d.f.=15.33, p=0.003), (B) decreased mean swim speed (two-tailed *t*-test, *t*=3.26, d.f.=10.88, p=0.008), and (C) decreased thigmotaxis (*t*-test, *t*=2.47, d.f.=12.86, p=0.028), in comparison to wild-type siblings. N= 9 wild-type and 9 GR mutant fish, 1 experiment.

(D, E) GR mutant adult males (two-tailed *t*-test, *t*=-3.26, d..f=4.05, p=0.03, N= 5 each, across 2 independent experiments) and 5 dpf larvae (two-tailed *t*-test, *t*=-3.30, d.f.=13.31, p=0.006, N= 12 pooled samples per group, across 3 independent experiments) have elevated baseline whole body cortisol levels compared to their wild-type siblings. All plots show mean +/- s.e.m. *, p<0.05, **, p<0.01, ***, p<0.001.

Disease Models & Mechanisms • Supplementary information

Table S1. Identification of differentially methylated CpGs using Whole Genome Bisulfite Sequencing and bsseq software. Methylation calls for CpGs were made using Bismark v0.15.0. Regions that were differentially methylated in GR mutants were detected using the bsseq R package. 249 differentially methylated CpG sequences were identified using bsseq. Genes were ranked were ranked by the areaStat parameter, which is the sum of the *t*-statistics for each CpG. DMRs with a larger number of CpGs exhibited greater areaStat values than DMRs with a smaller number of CpGs (Figure 3C). 142 DMRs (57%), exhibited hypermethylation of CpGs within the DMR in GR mutant (n=2) compared to wild-type (n=2) brain samples (denoted "hyper"). For the remaining 107 DMRs (43%), the CpG(s) were hypomethylated in the GR mutant compared to wild-type brain samples (denoted "hypo"). Each DMR was annotated with information about their proximity to transcription units.

Click here to download Table S1

Table S2. Gene Ontology analysis of GR DMRs. List of gene ontology biological process terms identified from the 249 DMRs using the Princeton GO term finder. 59 GO terms were detected with a p value cut-off of 0.05.

Click here to download Table S2

Table S3. Genes exhibiting significant differential expression in wild-type and GR mutant adult brain transcriptomes ranked according to adjusted p-value (p < 0.05). Raw count data was normalised with the DESeq2 function that calculates a normalisation factor using a median-of-ratios methods. A Wald test was used for hypothesis testing when performing differential expression analysis though which wild-types and mutant counts were compared. A Benjamini-Hochberg correction was used to correct for multiple comparisons (FDR cut-off <0.05). Genes were ranked based on their p-adjusted/FDR corrected p-values.

Click here to download Table S3

Table S4. Gene Ontology analysis for Biological Process terms linked to GR function using GOrilla. 32520 ENSDARG genes were ranked according to their adjusted p-value for significant differential expression in wild-type and GR mutant adult brain transcriptomes. Setting the threshold p-value for GO term assignment to $< 10^{-5}$ identified 19 Biological Process terms linked to the most significantly differentially expressed genes. Genes with roles in **chaperone-mediated protein folding** exhibited the most significant differential expression in wild-type and GR mutant adult brain samples (20 genes, p-value 8.65×10^{-12}, 6.04-fold enrichment), followed by genes with roles in **circadian regulation of gene expression** (14 genes, p-value 1.47×10^{-9}, 8.44-fold enrichment), **circadian rhythm** (18 genes, p-value 2.55×10^{-9}; 6.17-fold enrichment), and **regulation of circadian rhythm** (13 genes, p-value 3.64×10^{-9}; 8.74-fold enrichment). The table lists an additional 15 GO terms with p-values $< 10^{-5}$.

Click here to download Table S4

Table S5. Gene Ontology analysis for Molecular Function terms using GOrilla. 32520 ENSDARG genes were ranked according to their adjusted p-value for significant differential expression in wild-type and GR mutant adult brain transcriptomes. Setting the threshold p-value to $< 10^{-5}$ identified 4 Molecular Function terms linked to the most significantly differentially expressed genes. Genes with roles in **heat shock protein binding** exhibited the most significant differential expression in wild-type and GR mutant adult brain samples (16 genes, p-value 1.17×10^{-6}, 4.43-fold enrichment, followed by **protein serine/threonine kinase activity** (35 genes, p-value 1.67×10^{-6}, 2.6-fold enrichment), **transcription regulator activity** (145 genes, p-value 6.28×10^{-6}, 1.49-fold enrichment) and **unfolded protein binding** (22 genes, p-value 7.29×10^{-6}, 3.11-fold enrichment).

Click here to download Table S5

Disease Models & Mechanisms • Supplementary information

Table S6. Human diseases associated with human orthologues of differentially expressed genes in GR adult brain. The list of associated diseases was generated using the disgenet2r package (v7.0) and comparing to all DisGeNET databases. 678 diseases, symptoms or syndromes were found to be associated with GR-dependent differentially expressed genes (adjusted FDR, $p < 0.05$).

Click here to download Table S6

Table S7. Mental disorders associated with human orthologues of differentially expressed genes in GR adult brain. The list of mental disorders was generated using the disgenet2r package (v7.0). 26 mental disorders were found to be associated with genes exhibiting differential expression in wild-type and GR mutant adult brain samples (adjusted FDR, $p < 0.05$).

Click here to download Table S7

Table S8. Primer sequences used in BisPCR^2 analysis.
Samples subjected to BisPCR^2 analysis were PCR amplified twice. The first round of PCR utilised a DMR target region-specific primer pair, which also included an 18-bp overhang. The second round of PCR utilised barcoding primers which were designed to bind to the overhang created in PCR round one.

Click here to download Table S8
